# STRIDE: Species Tree Root Inference from Gene Duplication Events

**DOI:** 10.1093/molbev/msx259

**Published:** 2017-09-26

**Authors:** David M Emms, Steven Kelly

**Affiliations:** 1 Department of Plant Sciences, University of Oxford, Oxford, United Kingdom

**Keywords:** phylogenetics, phylogenomics, tree inference, gene duplication

## Abstract

The correct interpretation of any phylogenetic tree is dependent on that tree being correctly rooted. We present STRIDE, a fast, effective, and outgroup-free method for identification of gene duplication events and species tree root inference in large-scale molecular phylogenetic analyses. STRIDE identifies sets of well-supported in-group gene duplication events from a set of unrooted gene trees, and analyses these events to infer a probability distribution over an unrooted species tree for the location of its root. We show that STRIDE correctly identifies the root of the species tree in multiple large-scale molecular phylogenetic data sets spanning a wide range of timescales and taxonomic groups. We demonstrate that the novel probability model implemented in STRIDE can accurately represent the ambiguity in species tree root assignment for data sets where information is limited. Furthermore, application of STRIDE to outgroup-free inference of the origin of the eukaryotic tree resulted in a root probability distribution that provides additional support for leading hypotheses for the origin of the eukaryotes.

## Introduction

**Fig. 1.**
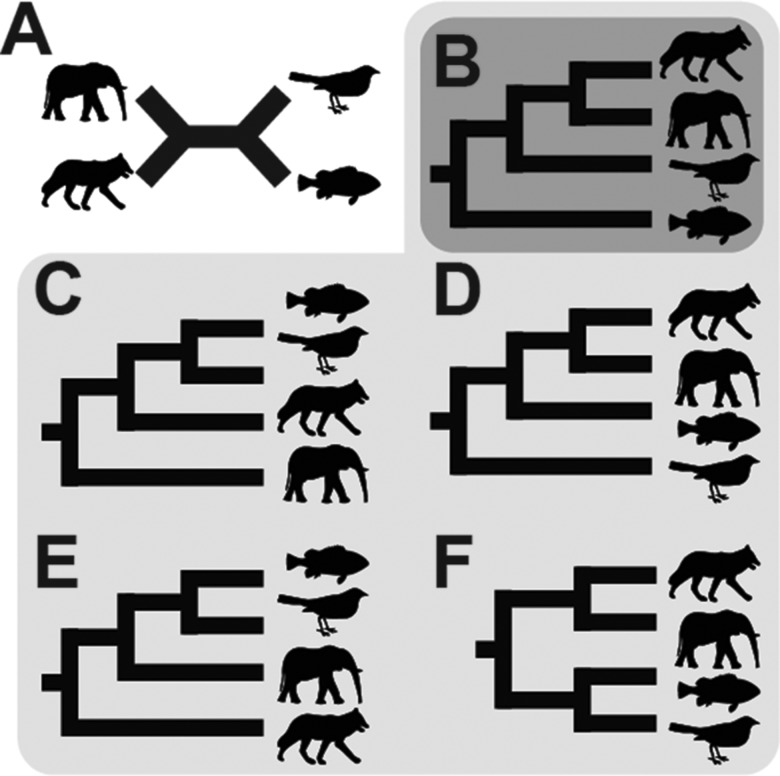
Possible roots for a four-taxa species tree. (*A*) Unrooted species tree for four species: elephant, wolf, fish, and bird. (*B*) The correct rooting of the species tree. (*B*–*F*) The five possible rooted species trees for the unrooted species tree in *A*.

The rooting of a phylogenetic tree is critical for the correct interpretation of the tree. For example, the phylogeny for four species (fig. 1*A*) has five possible roots (fig. 1*B*–*F*) and each of the different roots corresponds to a different hypothesis as to the evolutionary history of the species. For the presented tree, identifying a wrong branch as the root (e.g., fig. 1*E*) would lead us to conclude that elephants are more closely related to fish and birds than they are to wolves, even though we are using a tree with the correct topology. A species tree only gives the correct evolutionary relationships when rooted correctly (fig. 1*B*). Thus, it is of critical importance to our interpretation of relationships, and the evolutionary history of life on earth, that we have accurate methods of inferring the root of species phylogenies.

**Fig. 2.**
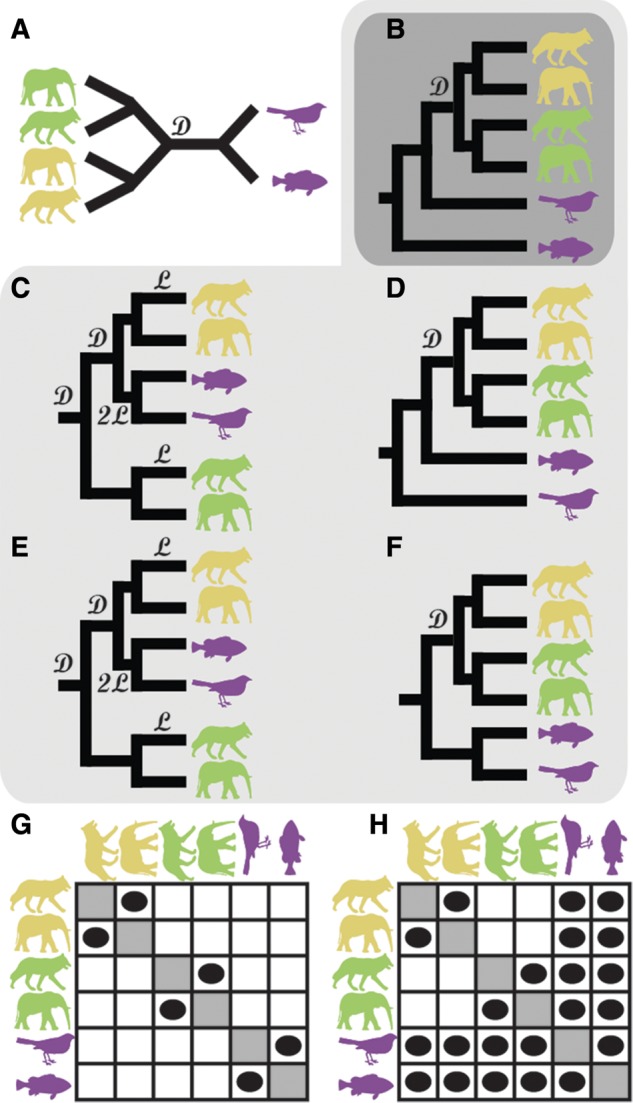
Orthologues inferred from gene trees depend on the root. (*A*) An unrooted gene tree corresponding to an orthogroup with a gene duplication event in the common ancestor of wolf and elephant. Genes from each species are represented by an image of the species. (*B*–*F*) The most parsimonious rootings of the gene trees (fewest duplications and losses) for each of the five roots of the species tree, as shown in figure 1*B*–*F*. D—gene duplication event, L—gene loss event. (*G*) Orthologues inferred from the incorrect trees *C* and *E*. (*H*) Orthologues inferred from the correctly rooted tree B and also the close to correctly rooted trees *D* and *F*.

Correct species tree rooting is also of critical importance for the inference of orthology relationships between genes. Given an unrooted gene tree (fig. 2*A*), knowledge of the correct branching order of the species tree (fig. 1*B*) is required to correctly root the gene tree (fig. 2*B*). An incorrect rooting of the species tree (fig. 1*C*–*F*) leads to an incorrect inference of the root of the gene tree (fig. 2*C*–*F*), and thus incorrect identification of orthologous genes (fig. 2*G*–*H*). Therefore, our ability to compare the biology of species, through comparisons between orthologous genes, is reliant on accurate methods of inferring the root of species phylogenies.

Although correct root placement is essential for our ability to interpret phylogenies, almost all models of sequence evolution used for tree inference are time-reversible and produce unrooted phylogenetic trees. In order to identify the root of a phylogeny extra information is required, usually knowledge of an extra species that is a suitable (i.e., closely related) outgroup for the set of species for which the root is unknown. However, outgroup choice is a common source of error in phylogenetic tree inference, with distantly related outgroups leading to inaccurate root placement and distortion of the phylogeny due to long branch attraction (Felsenstein 1981; Berger et al. 2011). Although time-irreversible models of sequence evolution exist, they do not provide a method for accurately inferring the direction of time in a tree (Huelsenbeck et al. 2002; Williams et al. 2015). To address this issue, methods have been developed that can simultaneously infer rooted species and gene trees (Boussau et al. 2013). However, these methods are computationally expensive and do not scale well to moderate or large species sets. Similarly, methods have been developed to root trees by minimizing a duplication and loss reconciliation cost (Chen et al. 2000; Gorecki and Tiuryn 2007; Gorecki et al. 2013). However, these require a rooted species tree for the reconciliation process.

“Duplicate gene rooting” has also been proposed as an alternative method for rooting species trees (Donoghue and Mathews 1998; Simmons et al. 2000). The conceptual basis for this approach is that gene duplication events are time-irreversible, unlike character substitution, and thus indicate the direction of time on the species tree. Specifically, every node in an unrooted, binary gene tree has three branches incident upon it. If the node is a speciation node then any of the three incident branches could be the edge in the direction of the root, with the other two being in the opposite direction. Thus, speciation nodes are uninformative about the direction of time along the tree. For a duplication node, however, the symmetry is broken. Two of the edges will correspond to the two copies of the gene postduplication, whereas the third edge will correspond to the gene preduplication and thus point towards the root of the tree (fig. 2*A*, node marked “D”). In the case of this example tree, it can be inferred that the root of the species tree must be outside of the subtree containing elephant and wolf. In an idealized case (with no effects such as incomplete lineage sorting or lateral gene transfer) the two postduplication branches can be distinguished from the preduplication branch as the postduplication branches contain genes from overlapping species sets. Furthermore, these species sets will be identical if there has been no gene loss or horizontal gene transfer, and the topology of the duplicate subtrees will recapitulate the species tree topology. Thus, if gene duplication nodes can be accurately identified in an unrooted gene tree, then the direction of time can be ascertained for all branches in the postduplication subtrees. The direction of time on these branches determines the direction of time on the corresponding branches of the species tree, and multiple gene duplication events can be aggregated to determine the direction of time across the whole species tree, thus revealing the location of the root.

Here, we present STRIDE, a novel algorithm for Species Tree Root Inference from gene Duplication Events. STRIDE identifies sets of well-supported gene duplication events from sets of unrooted gene trees, and analyses these events to infer a probability distribution over an unrooted species tree for the location of the true root. We show that STRIDE correctly identifies the community-accepted root of the majority of species trees. Additionally, we demonstrate that STRIDE effectively captures uncertainty in root placement when data is limited or conflicting. Finally, we demonstrate the utility of STRIDE to challenging phylogenetic problems by providing an outgroup-free root analysis of the origin of the eukaryotes.

## New Approaches

Here, we present a novel approach to rooting a species tree. Instead of requiring the inclusion of an outgroup species that is already known to be at the root of the species tree, STRIDE roots a species tree with no a priori information as to the location of the root. This is achieved through the identification of well-supported duplication events in a set of unrooted gene trees and from within the clade of species being studied. This removes the need for an outgroup and avoids problems that can arise due to long-branch attraction effects caused by an outgroup species.

## Results

### STRIDE Identifies the Correct Root of Species Trees Given Simulated Gene Tree Data Sets

The ability of STRIDE to correctly infer the root of a known species tree was tested using three published, simulated gene tree data sets. The first data set consisted of 2,000 simulated gene trees from 40 species with heterogeneous rates of gene duplication and loss within trees (Boussau et al. 2013). The second and third data sets consisted of 12,000 gene trees from 12 species and 7,500 gene trees from 17 species, respectively (Rasmussen and Kellis 2012). These two data sets were similar to the first data set but also incorporated incomplete lineage sorting generated using a range of effective population sizes. Since incomplete lineage sorting can lead to misidentification of gene duplication and loss events these latter two data sets provided a good test of STRIDE’s robustness in the face of gene-tree/species-tree incongruence. For all three simulated data sets, STRIDE correctly inferred the root of the species tree and assigned it a probability of 100% (table 1 and supplementary file S1 and fig. S1–S3, Supplementary Material online). Thus for these simulated data sets the method performed well.

**Table 1.** Summary of Data Sets and Results.

Group	Species	Gene Trees	Informative Dups^a^	Number of Conflicting Dups^a^	Number of MP Roots	Correct MP Root	Probability for MP Root (%)	Probability for Correct Root (%)
Metazoa (sim^b^)	40	2,000	664	0	1	Yes	100.0	100.0
Drosophila (sim^b^)	12	12,000	1,360	1	1	Yes	100.0	100.0
Primates (sim^b^)	17	7,500	1,593	0	1	Yes	100.0	100.0
Birds	47	14,454	51	0	1	No	15.0	2.0
Flies (Diptera)	7	11,688	279	11	1	Yes	100.0	100.0
Fish	11	16,520	650	7	1	Yes	100.0	100.0
Fungi	21	9,325	419	1	1	Yes	100.0	100.0
Hymenoptera	5	9,157	108	7	1	Yes	100.0	100.0
Kinetoplastids	16	9,731	76	4	1	Yes	55.0	55.0
Laurasiatheria	14	1,5804	135	7	1	No	100.0	0.0
Metazoa	21	13,017	2,065	0	1	Yes	48.0	48.0
Nematoda	7	8,392	93	2	1	Yes	100.0	100.0
Primates	11	19,096	117	11	1	Yes	8.0	8.0
Rodents	7	15,485	22	6	1	No	9.0	0.5
Plants	42	28,356	7,761	3	1	Yes	100.0	100.0
Eukaryotes	45	16,770	2,316	0	25	—	—	—

Total simulated	69	21,500	3,617	1	—	3	—	—
Total biological	254	187,795	14,092	59	—	9	—	—
Total	323	209,295	17,709	60	—	12	—	—

a Dups = duplications.

b sim = simulated.

Although there are no comparable methods for inferring the probability distribution for the root of a species tree from gene duplication events, there are methods that can identify gene duplication events in an unrooted gene tree if a rooted species tree is provided. It should be noted that these methods need to know the solution to the problem STRIDE is trying to solve in order to identify gene duplication events. However, an analysis of the gene duplication event detection accuracy of STRIDE in the context of these methods is provided in supplementary file S1 and table S1, Supplementary Material online. On the three simulated data sets, STRIDE had an overall precision of 99.9% and a recall of 34.8%. By comparison, on the same data sets Notung and DLCpar_search achieved an overall precision of 21.0% and 43.0%, respectively, and an overall recall of 84.0% and 75.3%, respectively (supplementary file S1 and table S1, Supplementary Material online). Thus, the precision of STRIDE is high even in the absence of knowledge of the root of the species tree. The recall was lower than the other methods, however it was sufficient to unambiguously pinpoint the location of the root in all three cases (table 1 and supplementary file S1 and fig. S1–S3, Supplementary Material online).

### Application of STRIDE to Real Species Data Sets

Simulated data sets generally do not capture all the nuances and difficulties seen in real biological data sets. These nuances include errors in orthogroup inference, alignment inference, and gene tree inference. Thus to demonstrate the utility of STRIDE, a diverse range of groups of species were sampled from throughout the eukaryotic domain (table 1). This included every group of eukaryotes on Ensembl Genomes containing >4 genera (Yates et al. 2016). To expand this group of tests, additional sets of genomes were obtained for 47 Birds (Jarvis et al. 2014), 42 Green Plants (Goodstein et al. 2012) and 16 Kinetoplastids (Aslett et al. 2010). In total, this gave 12 species groups with varying levels of taxon sampling and with estimated divergence times ranging from c. 56 My for the Primates (dos Reis et al. 2014) to c. 1,500 My for the Green Plants (Parfrey et al. 2011). These species sets thus provided a diverse group with which to test the utility of STRIDE. Furthermore, for each of these species sets, there is an accepted consensus on the topology and location of the root of the species tree (supplementary file S1, Supplementary Material online). In all cases these topologies and root branches were assumed to be true when STRIDE’s performance was assessed. On average, across each of the simulated and real data set in this analysis STRIDE took ∼18 s to run using four cores of an Intel Core i7-4770 3.4 GHz CPU.

Orthogroups for each species set were inferred using OrthoFinder (Emms and Kelly 2015), and gene trees for each orthogroup were inferred using IQTREE v1.5.3 (Nguyen et al. 2015) from a multiple sequence alignment generated using MAFFT L-INS-I v7.305b (Katoh and Standley 2013). For each species set, STRIDE was run with a published unrooted species tree (without branch lengths) and the complete set of gene trees inferred from all orthogroups identified by OrthoFinder. The number species, gene trees, informative duplications, and other details are provided in table 1.

**Fig. 3.**
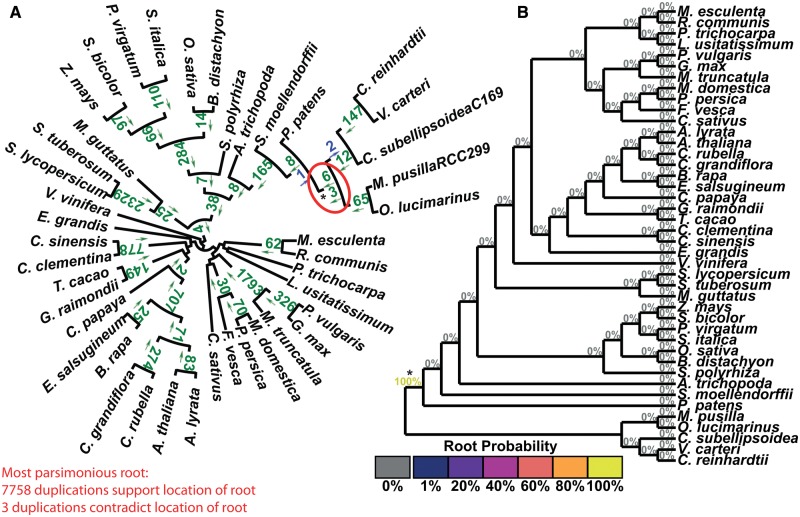
STRIDE analysis applied the set of plant gene trees. (*A*) Numbers of identified gene duplication events are marked on the branches they are observed on and arrows indicate the direction in which the duplication occurred. Gene duplication events are in agreement with the maximum parsimony root of the tree if the arrow points away from the root, and are in green. Those that disagree are in blue. The maximum parsimony root is circled in red and is in agreement with the correct root, marked with a *. (*B*) The probabilities for the location of the root calculated by STRIDE.

**Fig. 4.**
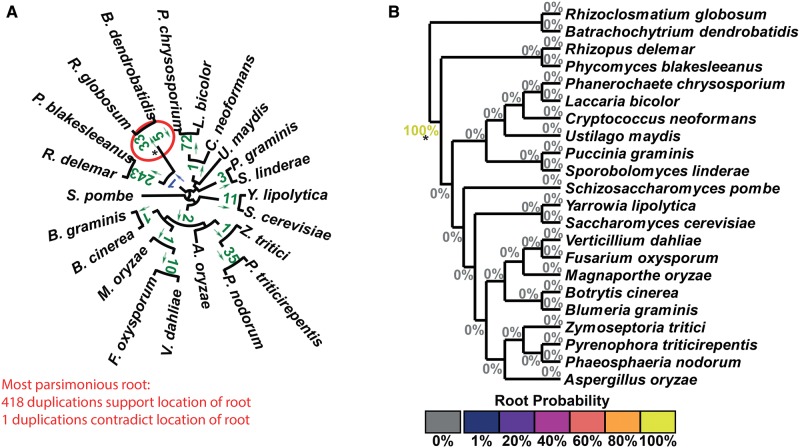
STRIDE analysis applied the set of fungi gene trees. (*A*) Numbers of identified gene duplication events are marked on the branches they are observed on and arrows indicate the direction in which the duplication occurred. Gene duplication events are in agreement with the maximum parsimony root of the tree if the arrow points away from the root, and are in green. Those that disagree are in blue. The maximum parsimony root is circled in red and is in agreement with the correct root, marked with a *. (*B*) The probabilities for the location of the root calculated by STRIDE.

**Fig. 5.**
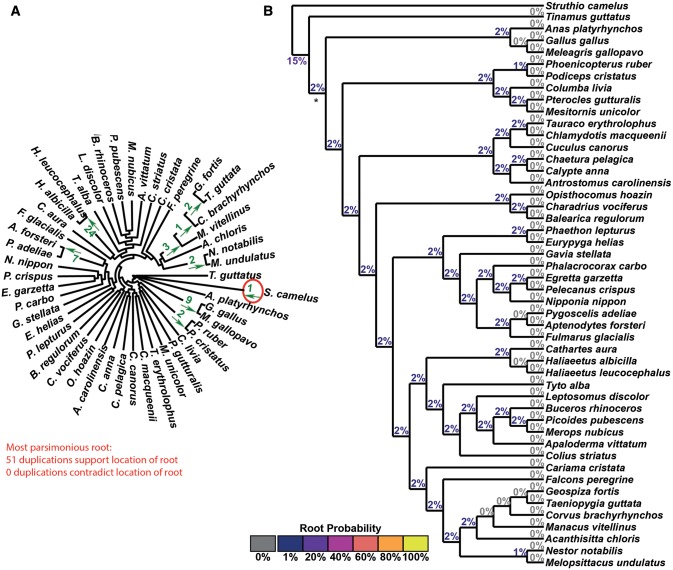
STRIDE analysis applied the set of Bird gene trees. (*A*) Numbers of identified gene duplication events are marked on the branches they are observed on and arrows indicate the direction in which the duplication occurred. Gene duplication events are in agreement with the maximum parsimony root of the tree if the arrow points away from the root, and are in green. Those that disagree are in blue. The maximum parsimony root is circled in red and is in agreement with the correct root, marked with a *. (*B*) The probabilities for the location of the root calculated by STRIDE, cultured according to the displayed heat map.

In all 12 test cases, there is a single maximum parsimony root. In nine of the 12 tests this root agreed with the accepted root of the species set (table 1). Figures 3–5 present the results of the STRIDE analysis applied to the plant, fungi, and bird data sets. These data sets correspond to the largest, median, and smallest number of informative duplications per species identified by STRIDE. The results for the remaining data sets can be found in supplementary file S1 and figures S4–S12, Supplementary Material online. For the plant data set, sufficient gene-duplication events were identified for the probability model to assign a probability of 100% to the accepted root separating the algae from the land plants (Ruhfel et al. 2014, 14: 23) (fig. 3). A probability of 100% was also assigned for the correct root in the fungi, even though fewer informative gene duplication events were identified (fig. 4 andtable 1). In both the plant and fungal data sets, STRIDE also identified substantial numbers of gene duplication events that support subclades within both species trees (figs. 3 and 4).

Although STRIDE identified the community-accepted root in 75% of the data sets, it failed to identify this root for the bird (fig. 5), rodent and Laurasiatheria (supplementary file S1 and figs. S11 and S12, Supplementary Material online) data sets. These three data sets had the smallest, second smallest and fourth smallest number of informative gene duplication events per species, respectively (table 1). In addition, while there were no conflicting gene duplication events in the bird data set, the rodent and Laurasiatheria data sets had the highest and fifth highest ratio of conflicting to informative duplications (table 1). Consistent with these observations, analysis of the factors affecting the accuracy of STRIDE revealed that root probability assignment was positively correlated with the number of informative duplications per species (*R*^2^ = 0.17, supplementary file S1 and fig. S13*A*, Supplementary Material online) and negatively correlated with the proportion of duplications which were in conflict (*R*^2^ = 0.24, supplementary file S1 and fig. S13*B*, Supplementary Material online). Furthermore, the proportion of conflicting duplications was negatively correlated with the number of species (*R*^2^ = 0.36, supplementary file S1 and fig. S13*C*, Supplementary Material online), suggesting increased taxon sampling facilitated more accurate identification of gene duplication events. Thus, the ability of STRIDE to detect the true root is affected by taxon sampling and the number of gene duplication events detected in the data set.

### STRIDE Provides Evidence for Location of the Root of the Eukaryotic Tree

**Fig. 6.**
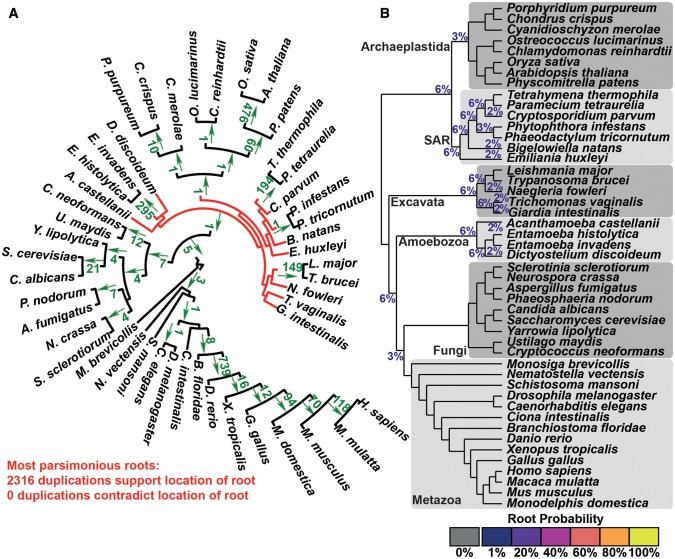
STRIDE analysis applied the set of Eukaryotic gene trees. (*A*) Numbers of identified gene duplication events are marked on the branches they are observed on and arrows indicate which block of the bipartition the duplicate genes occur in. None of the gene duplication events contradict each other. The maximum parsimony roots have red branches, the branches from which the root is excluded are black. (*B*) The probabilities for the location of the root calculated by STRIDE. Major groups of species are marked.

Given the performance of stride on the data sets outlined above it was assessed whether STRIDE could provide insight into one of the most contentious and difficult tree rooting problems in biology, the root of the eukaryotic tree (Burki 2014). Here, a set of 45 species that were distributed across the eukaryotic tree were selected. These were subject to orthogroup and gene tree inference as before and the complete set of 16,770 gene trees were submitted for analysis by STRIDE. This identified 2,316 gene duplication events excluding the root from (and supporting the monophyly of) major clades within the eukaryotes including the opisthokonta, fungi, metazoa, and achiplastida (fig. 6*A*). Duplication events supporting further subclades within these major groupings were also abundant (fig. 6*A*). In contrast, other major subclades including amoebazoa, the SAR supergroup, and the excavata, did not receive support from gene duplication events (fig. 6*A*). This lack of gene duplication events meant that STRIDE could not exclude the root of the species tree from the basal branches of these groups and thus could not provide evidence for or against the five most popular placements for the root of the eukaryotic tree (Burki 2014). This ambiguity in root assignment is represented effectively in the probabilities assigned to all putative root-spanning branches (fig. 6*B*).

### Discussion

STRIDE is an automated method for identifying and analyzing gene duplication events to infer the root of species trees. Through analysis of simulated and real data sets, we show how the performance of STRIDE is affected by data quantity, data conflict, and taxon sampling. Furthermore, we demonstrate that STRIDE is effective in identifying the root of species trees for the majority of species data sets and effectively captures the ambiguity in root assignment given the input data.

The aim of STRIDE is to infer a probability distribution over an entire species tree for the location of its root. This aim is different from algorithms that attempt to reconcile gene trees with species trees (Szöllősi et al. 2015) or model duplication and loss processes on a tree (Gorecki and Eulenstein 2014). STRIDE identifies and utilizes well-supported gene duplication events and does not evaluate gene loss events for the following reasons. First, gene trees can distinguish parallel duplication events on adjacent branches from a single shared duplication event, which is not possible for gene loss events. Second, the topology of the gene tree postduplication genes can be compared with the species tree to confirm the accuracy of the inference, this cannot be done with gene loss events. Third, most genomes are incomplete and vary considerably in the quality of their annotation leading to high rates of false positive gene loss (Veeckman et al. 2016; Dunne and Kelly 2017).

A major advantage of using STRIDE is that sets of species can be analyzed without the inclusion on an outgroup. This is potentially advantageous in situations where inclusion of an outgroup can affect the topology of gene trees inferred for the in-group species (Berger et al. 2011). Moreover, if the outgroup is distantly related to the in-group species then additional problems of long branch attraction can lead to incorrect root placement (Philippe et al. 2011; Kuck et al. 2012; Salichos and Rokas 2013). STRIDE is also suitable for large data set analysis and for situations where appropriate outgroups are not available. Although STRIDE as presented is a method for identifying the root of an unrooted species tree, the output from STRIDE can provide a wealth of useful information. For example, STRIDE identifies high confidence gene duplication events and maps these events to branches in a species tree. In the simulated data sets, only two of the 3,617 gene duplication events identified by STRIDE were incorrect. Similarly, in the real biological data sets only 60 of the 17,709 gene duplication events identified by STRIDE were conflicting and hence likely to be incorrect. As gene duplication events provide strong evidence for monophyly of the species that share the gene duplication event. STRIDE can be used to provide additional support to branches in a species tree that might be weakly supported by molecular sequence data. In this context, it is worth noting that STRIDE could also be used to evaluate support for alternative species-tree topologies by providing support for clades from gene duplication events.

The application of STRIDE to the eukaryotes was able to exclude the root of the eukaryotes from the opisthokonts and from a number of other groups, however STRIDE was unable to uniquely place the root as there were insufficient gene duplication events identified that could exclude the root from other portions of the tree. It is likely that poor taxon sampling for some of the groups (e.g., the amoebozoa and excavata), coupled with genome reduction associated with adaptation to parasitism in many of these species, impeded the discovery of these gene duplication events. With improved taxon sampling STRIDE may ultimately be able to provide further insight as to the location of the root of the eukaryotic tree. Furthermore, as STRIDE produces branch-level probabilities these could be combined with probabilities obtained from other analyses to perform a multi-data-type analysis of the origin of the eukaryotes.

In summary, STRIDE is a fast and effective method for genome scale phylogenetic analysis that can be used both to identify high confidence gene duplication events and identify the root of species trees without the requirement for an outgroup.

## Materials and Methods

### Problem Definition and Approach

A branch of an unrooted species tree corresponds to a bipartition that splits the tree’s taxa into two blocks. The presence of a well-supported gene duplication that respects the topology of the species tree is a synapamorphy that stipulates that the block in which the duplicates are found is a monophyletic clade. This synapamorphy identifies the direction of time along the branches within this monophyletic clade. The single exception to this is the branch in the unrooted tree corresponding to the root in which time flows in both directions. This is because the branch that spans the root in the unrooted species tree corresponds to two branches in the rooted species tree and both of its corresponding blocks are monophyletic clades (fig. 1*A* and *B*). The method presented here aims to identify this root branch by identifying and analyzing a set of well-supported gene-duplication events. The method identifies the set of well-supported gene duplication events contained within a set of user-supplied gene trees and uses these to infer the location of the root of the species tree. To express uncertainty in the case of limited data or data conflict, the method uses a probabilistic model of gene-duplication events to calculate a probability distribution across the branches of the species tree for the location of the root.

### Inference of Orthogroups and Gene Trees

For each species set, the protein sequence translations of representative gene models were downloaded from appropriate online databases. These protein sequences were then subject to orthogroup inference using OrthoFinder v1.1.4 (Emms and Kelly 2015). The resulting sets of protein sequence orthogroups were aligned using MAFFT L-INS-I v7.305b (Katoh and Standley 2013) and subject phylogenetic inference using IQTREE v1.5.3 (Nguyen et al. 2015). All methods used their default settings. Parallelization of MAFFT and IQTREE runs was done using GNU Parallel (Tange 2011). Alignments were viewed using AliView (Larsson 2014). Trees were viewed using Dendroscope (Huson and Scornavacca 2012) and drawn using the ETE library (Huerta-Cepas et al. 2016).

### Identification of Well-Supported Gene Duplication Events

Gene-duplication events are phylogenetically informative if they are observed in more than one species. Methods for identifying gene duplication events have previously been proposed that minimize a duplication and loss reconciliation of an unrooted gene tree with a rooted species tree (Chen et al. 2000; Gorecki and Tiuryn 2007). As STRIDE aims to identify the root of an unrooted species tree a novel method is proposed that does not require a rooted species tree as input. The notation used to describe the algorithm is defined in the following paragraphs. Additionally, a worked example of the algorithm with an accompanying explanatory figure is presented in supplementary file S1, text 1, and fig. S14, Supplementary Material online.

An unrooted tree is an unordered pair, T=(N,E), where N is the set of nodes and E is the set of undirected edges $\left\{n_{1},n_{2}\right\}$, $n_{1},n_{2}\in N$. The set of leaves, $L=\{n|degree\left(n\right)=1\}$, correspond to the taxa (genes or species) in the tree. Each edge in the tree corresponds to a bipartition, K, of the tree’s leaf set, splitting this set into two blocks, $K=B_{1}|B_{2}$, $B_{1},B_{2}\subset L$. For a rooted binary tree, we refer to the set of species below the (arbitrarily) left child node of the root by X and the species below the right child node of the root by Y. The sets of species below the grandchild nodes are $x_{1},x_{2},y_{1},y_{2}$, where $x_{1}\cup x_{2}=X$ and $y_{1}\cup y_{2}=Y$. For a gene tree, a set of genes is implicitly regarded as specifying another set, namely the set of species from which those genes come. The set of species in a subtree, t, is denoted S(t).

A few additional objects are required to describe the algorithm. The two blocks of a bipartition induce two disjoint subtrees. Let $\hat{T}(B)$ be the subtree of the species tree corresponding to block, B, rooted on the node separating this subtree from the other subtree. Let $\tilde{T}(n,e)$ be the rooted subtree of the gene tree “hanging” from e. Specifically, if e={n,n'}, then removing the edge e from the original tree gives two subtrees, one containing n and one containing n'. $\tilde{T}\left(n,e\right)$ is the subtree containing, and rooted on n'. Let B(S,t) be the smallest block of a bipartition of the species tree, t, such that S⊆B(S,t), for the set of species, S. Finally, let GC(t) be a function that returns the sets of taxa in the child and grandchild clades for the rooted subtree, t. Namely, X,Y,x1,x2,y1,y2=GC(t).

**Fig 7.**
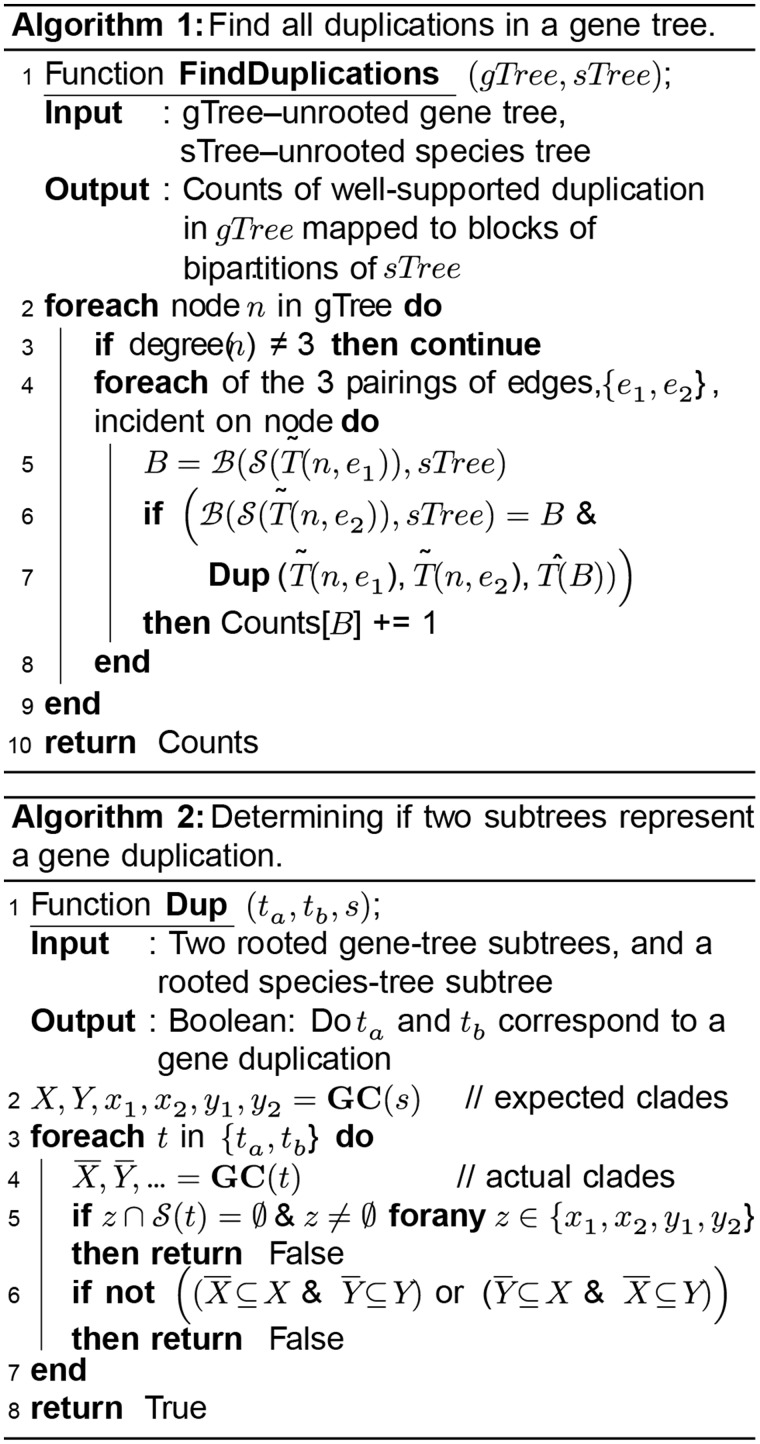
The STRIDE algorithm for identifying well-supported gene duplication events in an unrooted gene-tree. For details of the objects in the algorithm see “Methods: Identification of Well-Supported Gene Duplication Events.”

To identify all the well-supported gene duplication events in a set of unrooted gene trees, the algorithm “FindDuplications” (fig. 7) is run in parallel on the set of gene trees and the counts of the gene duplications are aggregated. The “FindDuplications” algorithm traverses an unrooted gene tree and in turn relies on the “Dup” algorithm (fig. 7) to determine if a pair of edges incident on a node in a gene tree correspond to a gene duplication event.

A gene duplication event for a block, *B*, of the species tree provides evidence that this block is a monophyletic clade. Partial gene loss subsequent to a gene duplication event can hamper the identification of the gene duplication event and the assignment of the event to the correct block of the species tree. The algorithm is made robust to this by mapping the observed set of species, S, in a subtree of a gene tree to the smallest block of the species tree, t, containing this species set, B(S,t) (fig. 7, Algorithm 1 line 5). Nodes with degree higher than three (unresolved polytomies) are excluded since they represent unresolved event in the gene tree (e.g., an amalgamation of several weakly supported bipartitions) and thus do not provide sufficiently strong evidence to accurately infer the gene history. To ensure that there is strong evidence for the gene-duplication event having occurred on the identified branch of the species tree, the presence of genes from each of the expected “grandchild” clades is required (fig. 7, Algorithm 2 line 5). Additionally, the local topology of each subtree postduplication is checked to ensure it matches the expected branching structure (fig. 7, Algorithm 2 line 6).

### Identifying the Maximum-Parsimony Root of the Species Tree

A gene duplication on an edge of an unrooted species tree with the duplicates observed in one of the blocks of the corresponding bipartition stipulates the direction of time for all edges in the subtree derived from that bipartition. Given a set of gene duplication events, the branch in the species tree that violates the fewest gene duplication events is identified as the maximum parsimony root. If multiple such branches exist then they are each identified as equally parsimonious. This method is similar to the plateau concept previously described (Gorecki et al. 2013).

### Probability Model for the Root of the Species Tree

For any given set of gene-trees, it is possible that errors in gene-tree inference will lead to false positive inference of gene duplication events that past the filtration criteria. To account for this, a probability model was developed for the location of the root of the tree given the set of (potentially conflicting) gene duplication events identified. The model consisted of two parts. The first part, the branch-level model, calculated the probability that a branch was the root given only the duplications observed in either direction along that branch. The second part, the tree-level model, aggregated all duplications observed across all branches of tree to give the final probability distribution for the location of the root taking into account all information obtained from all gene duplication events observed across the tree.

At the branch-level, the set of gene duplication events identified on that branch are modeled by two Poisson processes, one giving rise to true positive gene duplications and the other to false positive duplications. On a given branch, i, of a species tree, *m_i_* duplications are observed that support time flowing in one direction along the branch, ←, and *n_i_* duplications supporting time flowing in the opposite direction, →. The set of duplications on branch i is then written, $d_{i}=\langle m_{i},n_{i}\rangle$, and *D* is the set of duplications observed on all branches of the species tree, $D=\{d_{1},d_{2},\ldots ,d_{b}\}$.

Let $o_{i}\in \{\to ,\leftarrow ,root\}$ be the orientation of the branch i of the species tree and let $o_{j}^{\left(i\right)}\in \{\to ,\leftarrow ,root\}$ be the orientation of the branch j that would be implied by the root of the tree being branch i. The final tree-level probability distribution $P\left(o_{i}=root|D\right)$ takes into account the complete set of duplications, D, observed on all branches of the tree rather than just the duplications, $d_{i}$, observed on a single branch:

(1) $$P\left(o_{i}=root|D\right)=\frac{\prod_{j}P\left(o_{j}^{\left(i\right)}|d_{j}\right)}{\sum_{k}\prod_{j}P\left(o_{j}^{\left(k\right)}|d_{j}\right)}$$

That is, the probability distribution for the root given all the gene duplication events on the tree can be expressed in terms of the probabilities for the orientation of each branch given only the gene duplications on that branch; $P\left(\to |d_{i}\right)$, $P\left(\leftarrow |d_{i}\right)$ and $P\left(root|d_{i}\right)$.

### Poisson Model for Gene Duplications

To calculate $P\left(o_{i}|d_{i}\right)$ the duplications observed on a branch are modeled as arising from two Poisson processes. One process describes the number of true positive duplications (corresponding to the actual direction of time along the branch) and the other describes the number of false positive duplications. Let α be a parameter giving the relative frequency of false positives to true positives across all branches of the tree. Then m∼Po(λ) and n∼Po(αλ), where λ is the expected number of true positives on the branch. We set the total expected number of duplications on the branch from the two Poisson processes to match the actual number observed, *N*. Thus, λ=N/(1+α). The relative rate of false positives to true positives across the whole tree can be estimated from the number conflicting duplications given the maximum parsimony root of the tree. So as not to reward contradictory duplications by creating an expectation for them, we take α to be one tenth of the ratio of the observed conflicting to nonconflicting duplications of the maximum parsimony root. In almost all cases, however, the value of α had no discernible effect on the final probabilities of the model (supplementary file S1 and fig. S15, Supplementary Material online).

Bayes’ rule gives

$$P\left(o_{i}|d_{i}\right)=\frac{P(d_{i}|o_{i})P(o_{i})}{P(d_{i})}$$

where $P\left(d_{i}\right)=\sum_{o\in \{\to ,\leftarrow ,r\}}P\left(d_{i}||o\right)P(o)$. The priors are given by Proot=1b and P→=P←=b-12b, whereb=2t-3 is the number of branches on an unrooted tree with t taxa. The probability mass function for the Poisson distribution immediately gives $P\left(d||\leftarrow \right)$ and P(d|→):

$$P\left(d||\leftarrow \right)=Po\left(m;\lambda \right)Po\left(n;\alpha \lambda \right)$$

$$=\frac{\lambda^{m}e^{-\lambda }}{m!}\frac{{\left(\alpha \lambda \right)}^{n}e^{-\alpha \lambda }}{n!}$$

and,

$$P\left(d||\to \right)=Po\left(n;\lambda \right)Po\left(m;\alpha \lambda \right)$$

$$=\frac{\lambda^{n}e^{-\lambda }}{n!}\frac{{\left(\alpha \lambda \right)}^{m}e^{-\alpha \lambda }}{m!}$$

The branch with the root is more complicated since it actually corresponds to two branches on the rooted tree we are attempting to recover. On these two branches time flows in opposite directions, away from a central root that separates them. We must allow for the $\langle m,n\rangle$ duplications on the branch to actually correspond to $\langle m-s,t\rangle$ duplications on one of the two branches and $\langle n-t,s\rangle$ on the other branch (with opposite orientation to the first). The number of false positive duplications, s and t, are unknown and therefore must be summed over. Similarly, the location of root could fall at any point along the length of the original branch. If the root were a fraction, x, along the length of the branch then the expected rate of false positive and true positive duplications on that fraction of the branch would be xλ and xαλ, respectively, whereas on the other branch the rates would be (1-x)λ and (1-x)αλ. Thus, integrating over the position of the root along the branch and summing over the distribution of the $\langle m,n\rangle$ duplications between true positives and false positives on the two resulting branches, we find:

(2) $$P\left(d||root\right)=\sum_{s=0}^{m}\sum_{t=0}^{n}\int_{0}^{1}{Po}^{T}\left(m-s;x\lambda \right){Po}^{F}\left(t;x\alpha \lambda \right){Po}^{T}\left(n-t;(1-x)\lambda \right){Po}^{F}\left(s;(1-x)\alpha \lambda \right)dx=\sum_{s=0}^{m}\sum_{t=0}^{n}B(m-s+t+1,n-t+s+1)\frac{\lambda^{m-s}e^{-\lambda }}{\left(m-s\right)!}\frac{{\left(\alpha \lambda \right)}^{n-t}e^{-\alpha \lambda }}{\left(n-t\right)!}\frac{\lambda^{s+t}\alpha^{s+2t-n}}{s!t!}$$

Where B(,)is the beta function.

The duplications observed in just one species are uninformative as to the location of the root and so should not affect the root probabilities produced by the model. As such, the branch model for terminal branches is modified to only model the number of inward duplications (those supporting the tree minus the species on the terminal branch as a monophyletic clade). The rates $\lambda_{Term,TP}$ and $\lambda_{Term,FP}$ are the observed true positive and false positive rates for inward duplications on the terminal branches for the maximum parsimony root. For the terminal branches, the branch model is:

$$P^{Term}\left(d||\leftarrow \right)=Po\left(m;\lambda_{Term,FP}\right)$$

and

$$P^{Term}\left(d||root\right)=Po\left(m;\lambda_{Term,TP}\right).$$

**Fig. 8.**
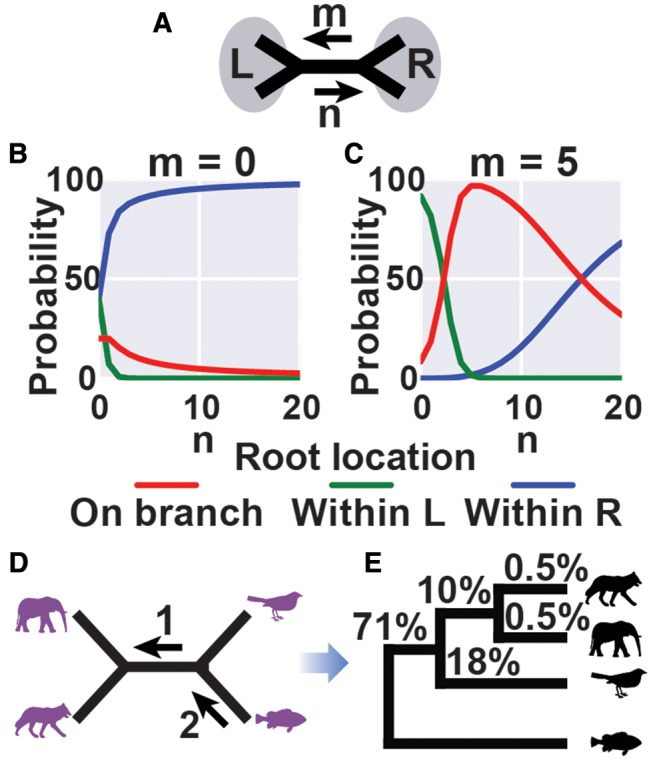
The branch-level probability model employed by STRIDE. These branch-level probabilities are used by the tree probability model to give the overall probabilities for the location of the root of the species tree. (*A*) A single branch in the tree with m/n duplications supporting L/R as monophyletic clades. (*B*) Branch-level model probabilities for position of the root with respect to the branch when m = 0 (the model only takes into account duplications on that branch). (*C*) As for B with m = 5. (*D*) Hypothetical total number of gene duplication events on the four species phylogeny. One gene duplication event is shared by elephant and dog and two are shared by elephant, dog, and bird. (*E*) The final tree-level model probabilities for the location of the root calculated by STRIDE taking into account all the gene duplication events on all branches in D.

The branch-level model takes into account only the duplications observed on a single branch and these probabilities feed into the tree-level model to give the final probabilities for the position of the root (fig. 8). The behaviour of the branch model is in good agreement with an intuitive understanding of the probabilities that should be assigned to the three possible orientations for a branch given the number of putative duplications observed in either direction (fig. 8*A*–*C*). The probability of time flowing to the left/right increases monotonically with the number of putative duplications supporting it. The probability of a branch being a root is highest when the number of putative gene duplications in both directions is the same. Finally, the probability of a branch being a root remains significantly above zero if there is any number of gene duplications in both directions (fig. 8*B* and *C*). This reflects the fact that putative gene duplications supporting the monophyletic nature of both blocks of a bipartition support that bipartition being the root. The fact that there could be a large difference in the number of gene duplications in one direction compared with the other due to different branch lengths on the two sides of the root is accounted for by integrating over the position of the root along the original root branch. Thus, the probability of a branch being a root is > 30% when there are 20 duplications in one direction compared with five in the opposite direction (fig. 8*C*). For comparison, the probability of the orientation of the branch being in the direction of the five duplications is vanishingly small (∼10^−13^). The branch-level probability model thus gives probabilities for each branch taking into account only the duplications observed on that branch. The final probabilities for the root of the tree, taking into account all duplications across the tree are then given by the tree-level model (equation (1), fig. 8*D* and *E*).

### Time-Complexity

For a set of gene trees containing N genes in total from n species, the identification of all well-supported gene duplication events can be achieved in time $O\left(nN\right)$ and the calculation of the probabilities in $O\left(\frac{N}{n}.N\right)$ as described in the following analysis.

Each gene tree can be analyzed using three traversals of the bipartitions of the gene tree. The first two are preprocessing steps that cache the sets of species either side of each edge in the tree while the third traversal identifies all well-supported gene duplications in the tree (in fact, the second and third traversals can be combined). The three traversals are as follows: With an arbitrary root of the tree, a postorder traversal is performed first to cache, for each edge, the set of species below that edge of the tree, a preorder traversal then caches the set of species above each edge using the data cached in the postorder traversal. This takes $O\left(Mn\right)$ for a single gene tree, where M is the number of genes in the species tree. For all gene trees this takes O(Nn). To identify the well-supported gene duplications a tree must be traversed again (visiting the edges in any order). For each edge the algorithm: Determines β, the smallest bipartition of the species tree containing the species (fig. 7, Algorithm 1, line 5) in average O(n). For each putative duplication the algorithm checks that at least one gene from each of the expected grandchild clades is present (fig. 7, Algorithm 2, line 5) in average O(n). For each putative duplication, the algorithms checks that each of the actual child clades are subsets of the expected child clades (fig. 7, Algorithm 2, line 5) in average O(n). Thus, the identification of all well-supported gene duplications events in a single gene tree is O(nM), and is O(nN) for all gene trees since the total number of edges in the set of all gene trees is O(N).

Having identified the well-supported duplications in all gene trees, the maximum parsimony root can be identified in O(n). In practice, calculating the final probabilities for the location of the root using Equations (1) and (2) is trivial. Although a naive evaluation of Equation (1) requires $O\left(n^{2}\right)$, it can be calculated in time O(n) (Felsenstein 2004). For Equation (2), assuming that the number of duplications per branch of the species tree is proportional to the number of gene families, Nn, then the time-complexity is $O\left(\frac{N}{n}.N\right)$.

### Testing Gene Duplication Event Identification Accuracy

The gene duplication event identification accuracy was examined on the three simulated data sets, for which the ground truth was known (Rasmussen and Kellis 2012; Boussau et al. 2013). STRIDE was run as normal using as input the unrooted species tree and the set of unrooted gene trees. For comparison two representative tree reconciliation methods, Notung (Chen et al. 2000) and dlcpar_search (Wu et al. 2014), were also run on the same data sets. The comparison was not exact since both of these reconciliation methods require a rooted species tree (information not available to STRIDE). Notung was run with the rooted species tree and unrooted gene trees using default parameters and the “–root” option, which roots the gene tree on the branch giving the lowest overall reconciliation cost. Dlcpar_search is a more sophisticated tree reconciliation program that aims to give higher precision inference of duplications and losses by also modeling deep coalescence so as to better explain incongruence between the gene tree and the species tree that can arise from incomplete lineage sorting. The program performs a heuristic search for a tree minimizing an overall duplication, loss and coalescence cost. As it also requires that the gene trees be rooted, the companion program “reconroot” was used to first root the gene trees (as recommended, private correspondence) on the branch giving the lowest reconciliation cost when only duplication and loss events are considered. The dlcpar_search method was then run on the rooted species tree and rooted gene trees using default parameters.

### Algorithm Implementation and Availability

STRIDE is implemented in python. Further information, use instructions, an example data set, and a standalone implementation of the algorithm is available under the University of Oxford Academic Use Licence at https://github.com/davidemms/STRIDE, last accessed October 4, 2017. The complete set of gene trees and species trees required to replicate this analysis are provided for download form the Zenodo research data archive at https://doi.org/10.5281/zenodo.581360, last accessed October 4, 2017.

## Supplementary Material

Supplementary Data
