# Supplementary material for: STRIDE: Species Tree Root Inference from Gene Duplication Events
Source: Mol Biol Evol. 2017 Sep 26;34(12):3267–78. doi: 10.1093/molbev/msx259 (PMC5850722; doi:10.1093/molbev/msx259)
Supplement: Supplementary Data [file msx259_supp.pdf]

Citations for the species trees used in this analysis

The topology of the species tree for each of the 12 datasets used in this analysis. The rooted versions of these trees were taken as true and used for evaluation of STRIDE. The unrooted version of these species trees were used as input for STRIDE.

Birds (Jarvis, E.D., Mirarab, S., et al. 2014), **Flies** (Wiegmann, B.M., Trautwein, M.D., et al. 2011), **Fish** (Betancur, R.R., Broughton, R.E., et al. 2013), **Fungi** (James, T.Y., Kauff, F., et al. 2006), **Hymenoptera** (Mao, M., Gibson, T., et al. 2015), **Kinetoplastids** (Seward, E.A. and Kelly, S. 2016), **Laurasiatheria** (Meredith, R.W., Janecka, J.E., et al. 2011), **Metazoa** (Dunn, C.W., Hejnol, A., et al. 2008), **Nematoda** (Zhou, Y. and Holmes, E.C. 2007), **Primates** (Perelman, P., Johnson, W.E., et al. 2011), Rodents (Meredith, R.W., Janecka, J.E., et al. 2011) and **Plants** (Bremer, B., Bremer, K., et al. 2009).

References

Betancur RR, Broughton RE, Wiley EO, Carpenter K, Lopez JA, Li C, Holcroft NI, Arcila D, Sanciangco M, Cureton Ii JC*, et al.* 2013. The tree of life and a new classification of bony fishes. PLoS Curr, 5.

Bremer B, Bremer K, Chase MW, Fay MF, Reveal JL, Soltis DE, Soltis PS, Stevens PF, Anderberg AA, Moore MJ*, et al.* 2009. An update of the Angiosperm Phylogeny Group classification for the orders and families of flowering plants: APG III. Bot J Linn Soc, 161:105-121.

Dunn CW, Hejnol A, Matus DQ, Pang K, Browne WE, Smith SA, Seaver E, Rouse GW, Obst M, Edgecombe GD*, et al.* 2008. Broad phylogenomic sampling improves resolution of the animal tree of life. Nature, 452:745-U745.

James TY, Kauff F, Schoch CL, Matheny PB, Hofstetter V, Cox CJ, Celio G, Gueidan C, Fraker E, Miadlikowska J*, et al.* 2006. Reconstructing the early evolution of Fungi using a six-gene phylogeny. Nature, 443:818-822.

Jarvis ED, Mirarab S, Aberer AJ, Li B, Houde P, Li C, Ho SYW, Faircloth BC, Nabholz B, Howard JT*, et al.* 2014. Whole-genome analyses resolve early branches in the tree of life of modern birds. Science, 346:1320-1331.

Mao M, Gibson T, Dowton M. 2015. Higher-level phylogeny of the Hymenoptera inferred from mitochondrial genomes. Mol Phylogenet Evol, 84:34-43.

Meredith RW, Janecka JE, Gatesy J, Ryder OA, Fisher CA, Teeling EC, Goodbla A, Eizirik E, Simao TLL, Stadler T*, et al.* 2011. Impacts of the Cretaceous Terrestrial Revolution and KPg Extinction on Mammal Diversification. Science, 334:521-524.

Perelman P, Johnson WE, Roos C, Seuanez HN, Horvath JE, Moreira MAM, Kessing B, Pontius J, Roelke M, Rumpler Y*, et al.* 2011. A Molecular Phylogeny of Living Primates. Plos Genet, 7.

Seward EA, Kelly S. 2016. Dietary nitrogen alters codon bias and genome composition in parasitic microorganisms. Genome Biol, 17.

Wiegmann BM, Trautwein MD, Winkler IS, Barr NB, Kim JW, Lambkin C, Bertone MA, Cassel BK, Bayless KM, Heimberg AM*, et al.* 2011. Episodic radiations in the fly tree of life. Proceedings of the National Academy of Sciences of the United States of America, 108:5690-5695.

Zhou Y, Holmes EC. 2007. Bayesian estimates of the evolutionary rate and age of hepatitis B virus. J Mol Evol, 65:197-205.

Supplemental Text 1

Prior to analysis of the gene trees, the unrooted species tree was analysed to determine the species sets in which genes would be expected to occur following a gene duplication event along any branch of the species tree. For each direction along each branch the sets of species in the child clades (X & Y) and in the grandchild clades (x_1, x_2, y_1 & y_2) immediately following that branch were identified (Fig. 8A). Then each gene tree was analysed in turn to identify well-supported gene duplication events within the gene tree as follows: each node, n, in the tree was considered in turn, if the node was an unresolved polytomy it was excluded as such nodes correspond to either a higher-order multiplication events (e.g. triplication) or an unresolved event in the gene tree (e.g. an amalgamation

of several weakly supported bipartitions). Each analysed node therefore had three branches incident on it, and any pair of branches could potentially represent duplicate genes (Fig. 8B). For each pair of branches, b_1 and b_2, the sets of species, S_1 and S_2, below each branch were used to identify the locations in the species tree corresponding to these branches in the gene tree. This was done by identifying the smallest block, B_i in the species tree that contains all the species in S_i (i=1,2), thus making the method robust in the case of subsequent gene loss (Fig. 8B). If there was more than one block satisfying this criteria, each of these possible blocks were considered. A node, n, was considered as a putative gene duplication event if $B_1=B_2$.

Nodes that were identified as putative gene duplication events were further examined to reduce the possibility that their existence or location had been misidentified due to errors in gene tree inference. The criteria were: 1) There must be at least one gene from each of the expected grandchild clades in both S_1 and S_2 (Fig. 8C). 2) The branching structure immediately after the gene duplication event on branches b_1 and b_2 must match the expected branching structure (Fig. 8D), i.e. the first node for each duplicate split the descendent species into the expected sets X and Y, or subsets thereof. Note that it would not be valid to check the topology to the level of grandchild clades in step 2 since this would fail to identify gene duplication events if there were also a subsequent gene duplication event one branch lower in the species tree. In this case, the observed grandchild clades would both be subsets of one of the expected child clades rather than grandchild clades. Steps 1 and 2 check that the observed clades are subsets of the expected clades (rather than requiring they be equal to) as this is necessary to make the method robust to subsequent gene loss events.

Supplemental Figure Legends

Supplementary Figures 1 -12

STRIDE analysis applied to the test datasets gene trees 1) Simulated metazoa dataset 2) Simulated fly dataset 3) Simulated primate dataset 4) Diptera gene trees 5) Fish gene trees 6) Hymenoptera gene trees 7) Kinetoplastid gene trees 8) Metazoa gene trees 9) Nematoda gene trees 10) Primate gene trees 11) Laurasiatheria gene trees 12) Rodent gene trees. A) Numbers of identified gene duplication events are marked on the branches they are observed on and arrows indicate which

block of the bipartition the duplicate genes occur in. Gene duplication events are in agreement with the maximum parsimony root of the tree if the arrow points away from the root, and are in green. Gene duplication events are in disagreement with the maximum parsimony root if the arrow points towards the root (thus suggesting that the indicated clade, that spans the marked root, is monophyletic) and are in blue. The maximum parsimony root is circled in red. B) The probabilities for the location of the root calculated by STRIDE coloured according to the displayed heat map. The correct root is marked with an asterisk.

Supplemental Figure S13

Factors affecting the accuracy of STRIDE. A) Number of duplications divided by number of species versus the probability assigned to the correct root. B) Number of duplications conflicting with the maximum parsimony root versus the probability assigned to the correct root. C) Number of conflicting duplications versus the number of species.

Supplemental Figure S14

Identification of well-supported gene duplication events for an example gene tree. Upper case letters M,N,O,P & Q are species, lower case m,n,o,p & q are genes from the corresponding species. A) The unknown, rooted species tree (left) and the observed, unrooted species tree (right). Black dot and arrow show the location of a single hypothetical gene duplication event on a branch with time flowing in the direction indicated by the arrow. The branch location and direction is uniquely identified by the block, B, of species whose common ancestor would have inherited the duplicate genes. The expected species in the child clades (X & Y) and grandchild clades (x_1, x_2 & Y) after this hypothetical duplication are highlighted with light/dark grey ellipses respectively. B) The unknown, rooted gene tree (left) and the observed, unrooted gene tree (right) for a hypothetical gene family with three gene duplication events (marked by *) and two gene loss events (grey, dotted line). The node currently being analysed is n and b_r is the current, tentative direction towards the root. For these n and b_r, b_1 and b_2 are analysed to see if they are well-supported gene duplication branches. Si is the set of species below branch b_i, B_i is the smallest block of a bipartition containing S_i (i=1,2) C) The check that genes from each of the expected grandchild clades are present on each duplicate branch D)

The check that the local topology for each duplication branch agrees with expected topology. U_i and V_i are the observed child clades on branch b_i. The observed child clades should not contain genes from any species not in the expected child clades

Supplemental Figure S15

The dependence of the probability for the root of species tree assigned by STRIDE on the parameter, α, that specifies the ratio of the expected number of false-positive duplications to true-positive duplications on a branch. For each plot, α ranges from one-tenth of the actual value used by STRIDE ($\alpha'/10$) to ten times the value used by STRIDE ($10\alpha'$).

Supplementary Figures

Fig. S1

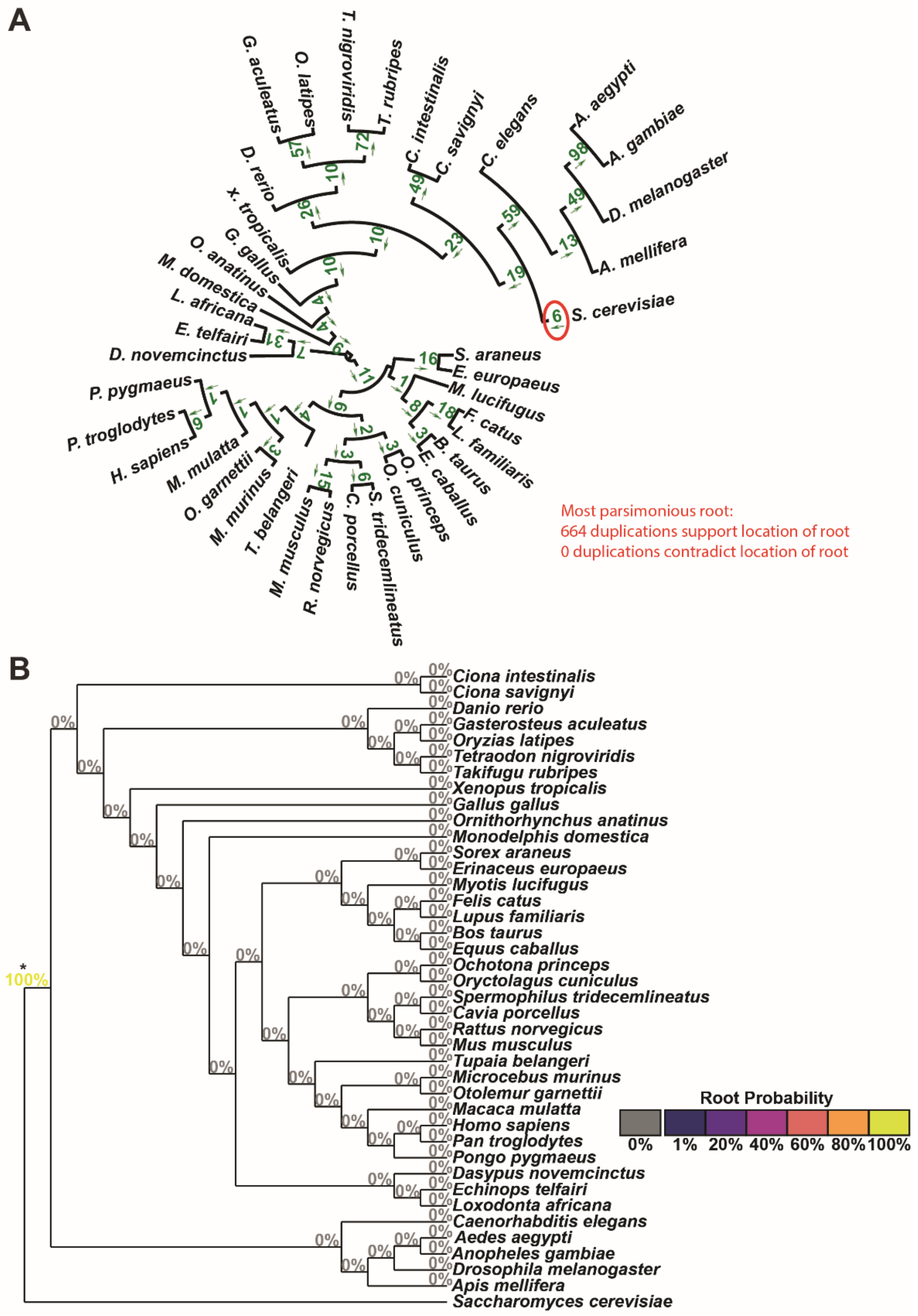

Fig S2.

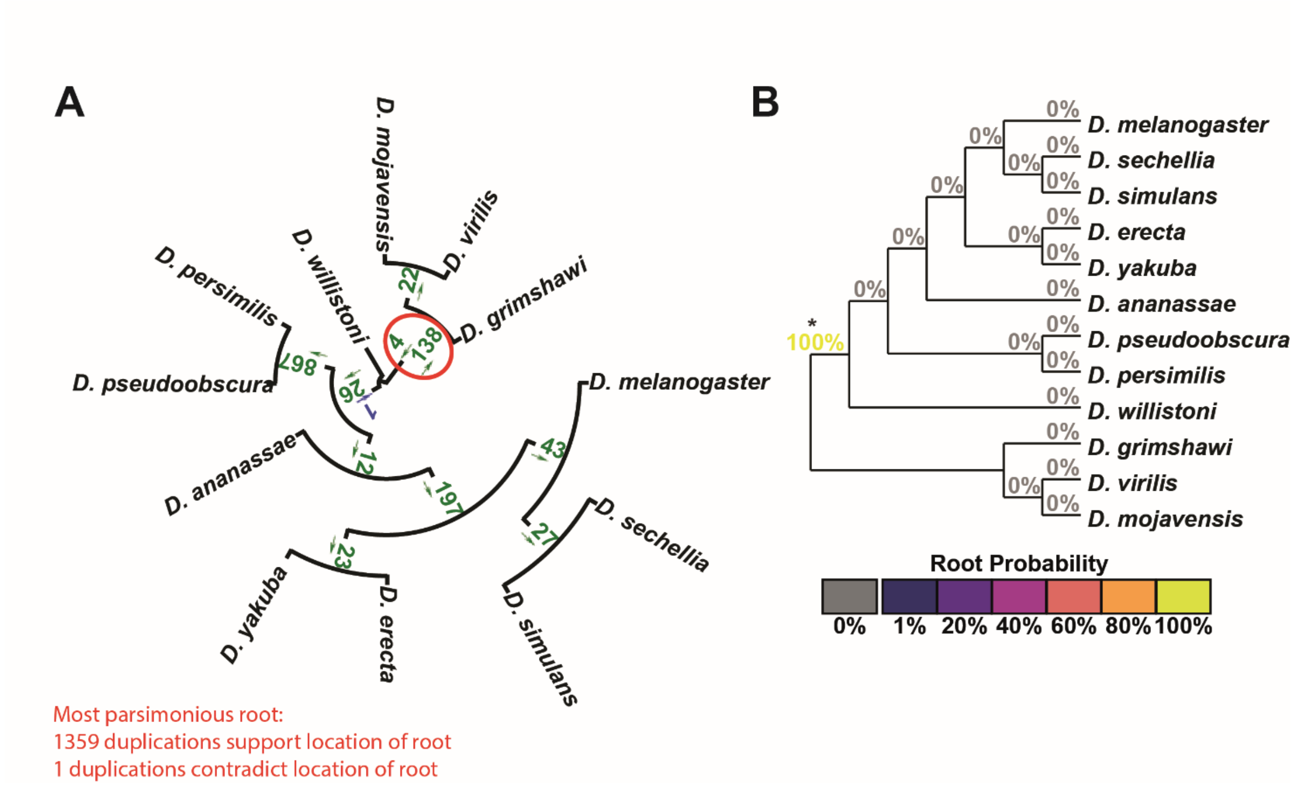
A
B
D. mojavensis
D. virilis
D. grimshawi
D. willistoni
D. persimilis
D. pseudoobscura
D. melanogaster
D. ananassae
D. sechellia
D. yakuba
D. erecta
D. simulans
Most parsimonious root:
1359 duplications support location of root
1 duplications contradict location of root
100%
0%
Root Probability
0%
1%
20%
40%
60%
80%
100%

Fig. S3

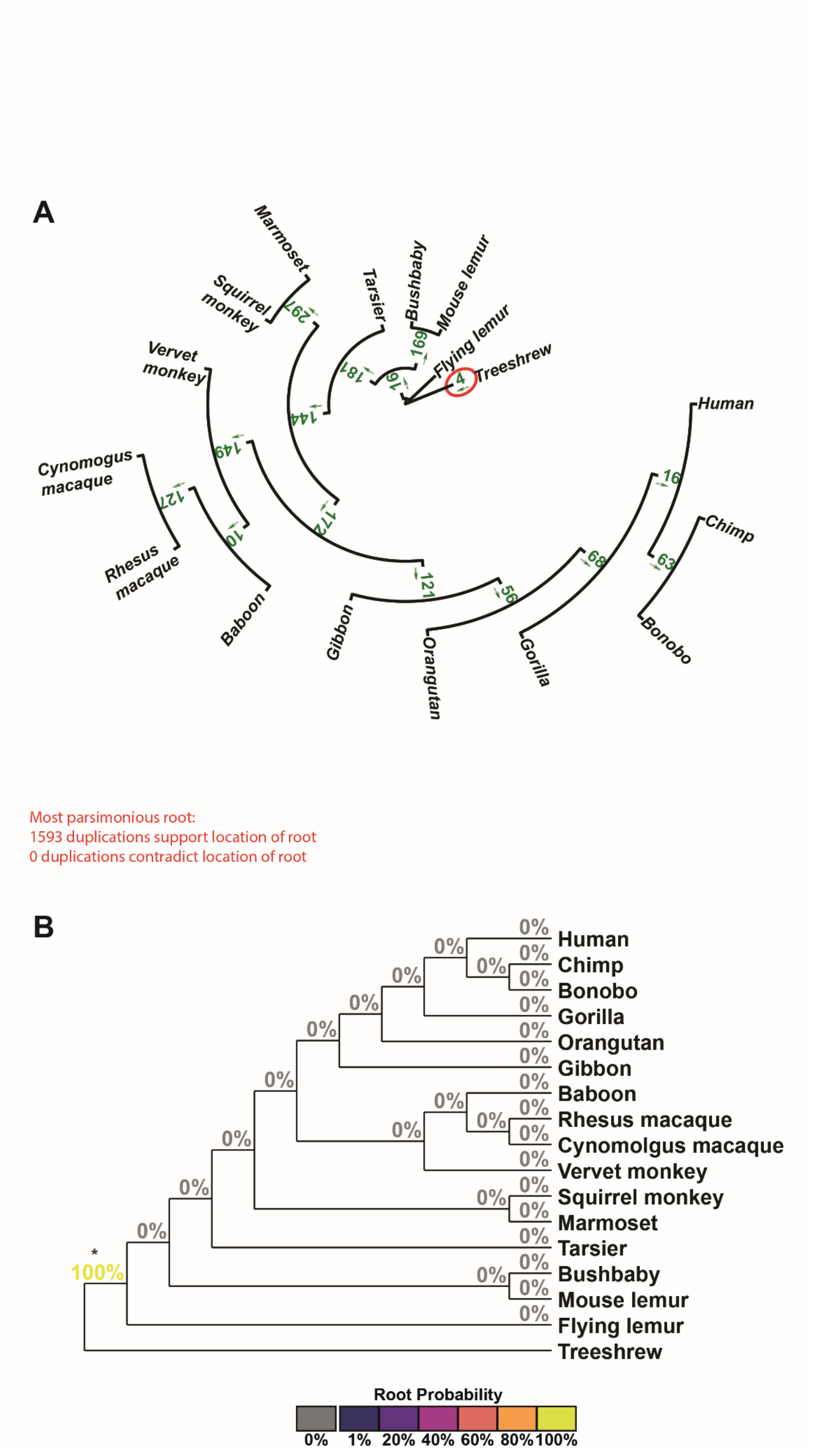

A
Marmoset
Squirrel monkey
Tarsier
Bushbaby
Mouse lemur
Flying lemur
Treeshrew
Vervet monkey
Cynomogus macaque
Rhesus macaque
Baboon
Gibbon
Orangutan
Gorilla
Bonobo
Chimp
Human
297
169
181
16
4
144
149
127
10
172
121
56
68
16
63
Most parsimonious root:
1593 duplications support location of root
0 duplications contradict location of root
B
Human
Chimp
Bonobo
Gorilla
Orangutan
Gibbon
Baboon
Rhesus macaque
Cynomolgus macaque
Vervet monkey
Squirrel monkey
Marmoset
Tarsier
Bushbaby
Mouse lemur
Flying lemur
Treeshrew
*
100%
0%
Root Probability
0%
1%
20%
40%
60%
80%
100%

Fig. S4

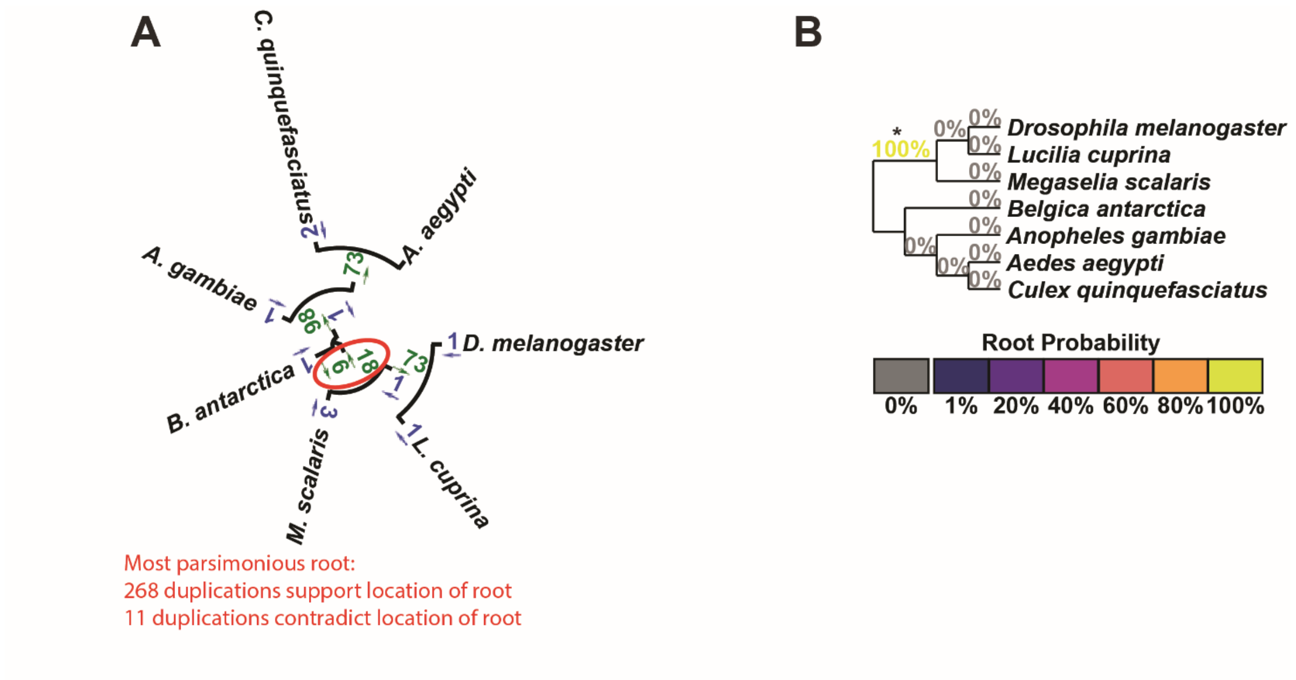

A
C. quinquefasciatus
A. aegypti
A. gambiae
B. antarctica
M. scalaris
L. cuprina
D. melanogaster
Most parsimonious root:
268 duplications support location of root
11 duplications contradict location of root
B
Drosophila melanogaster
Lucilia cuprina
Megaselia scalaris
Belgica antarctica
Anopheles gambiae
Aedes aegypti
Culex quinquefasciatus
Root Probability
0%
1%
20%
40%
60%
80%
100%

Fig. S5

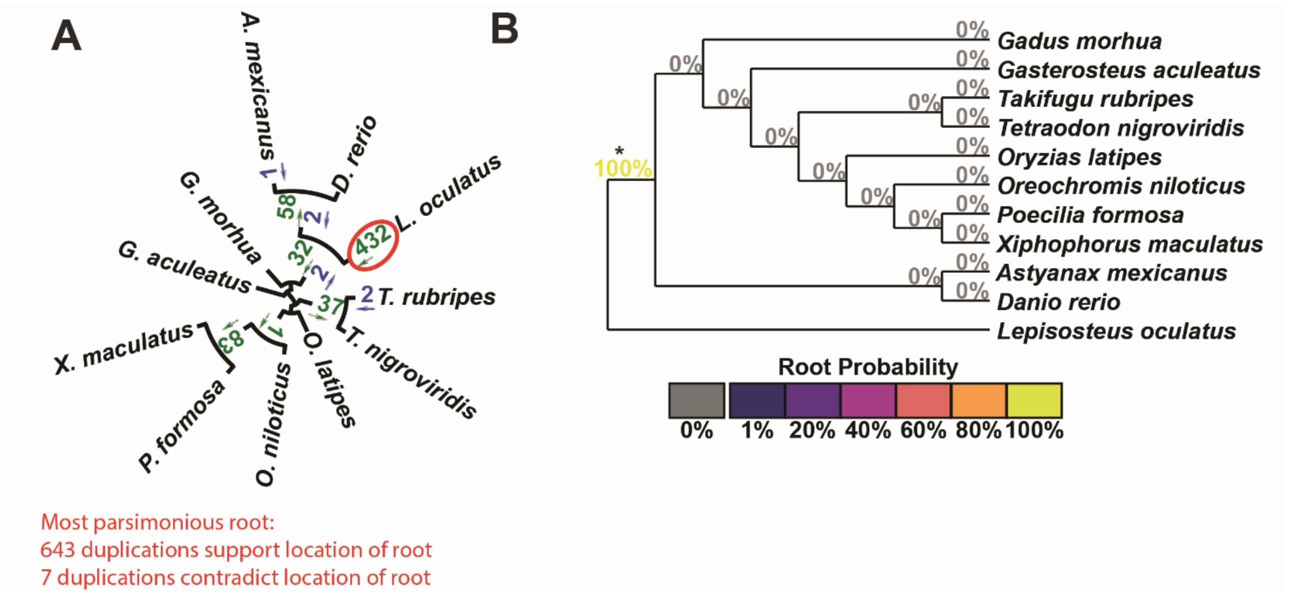
A
B
A. mexicanus
D. rerio
L. oculatus
G. morhua
G. aculeatus
T. rubripes
T. nigroviridis
O. latipes
O. niloticus
P. formosa
X. maculatus
1
58
2
432
32
2
37
2
38
1
Most parsimonious root:
643 duplications support location of root
7 duplications contradict location of root
Gadus morhua
Gasterosteus aculeatus
Takifugu rubripes
Tetraodon nigroviridis
Oryzias latipes
Oreochromis niloticus
Poecilia formosa
Xiphophorus maculatus
Astyanax mexicanus
Danio rerio
Lepisosteus oculatus
100%
*
Root Probability
0%
1%
20%
40%
60%
80%
100%

Fig S6

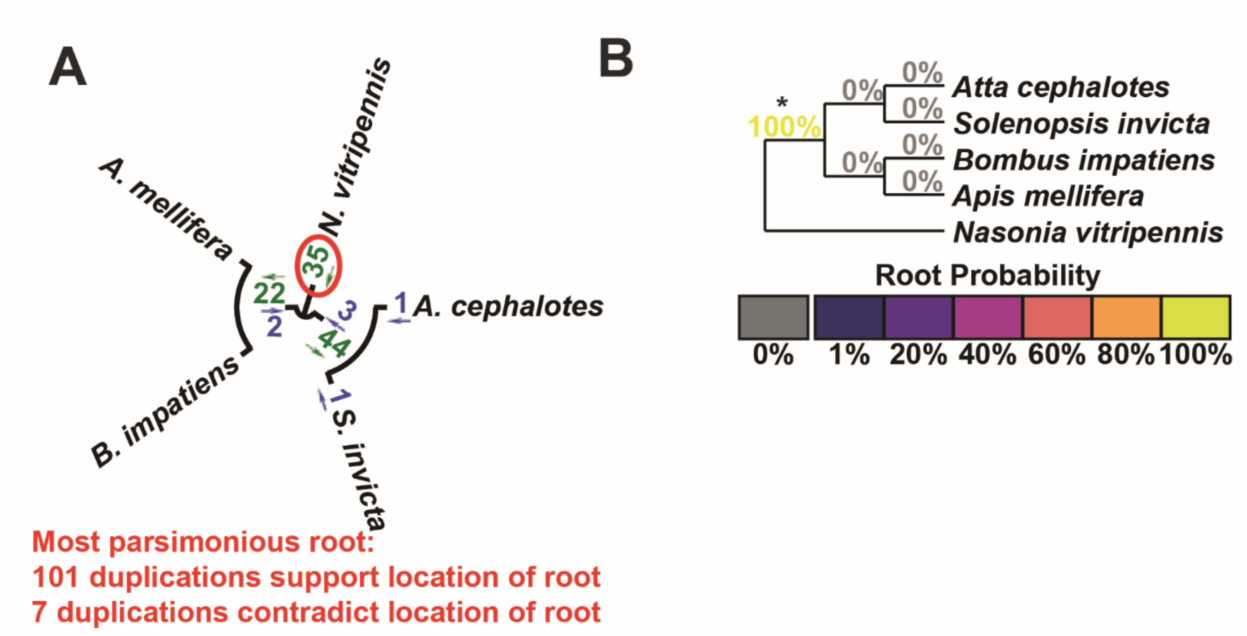
A
N. vitripennis
A. mellifera
35
22
2
3
1
A. cephalotes
44
B. impatiens
1
S. invicta
Most parsimonious root:
101 duplications support location of root
7 duplications contradict location of root
B
*
100%
0%
0%
0%
0%
0%
0%
Atta cephalotes
Solenopsis invicta
Bombus impatiens
Apis mellifera
Nasonia vitripennis
Root Probability
0%
1%
20%
40%
60%
80%
100%

Fig. S7

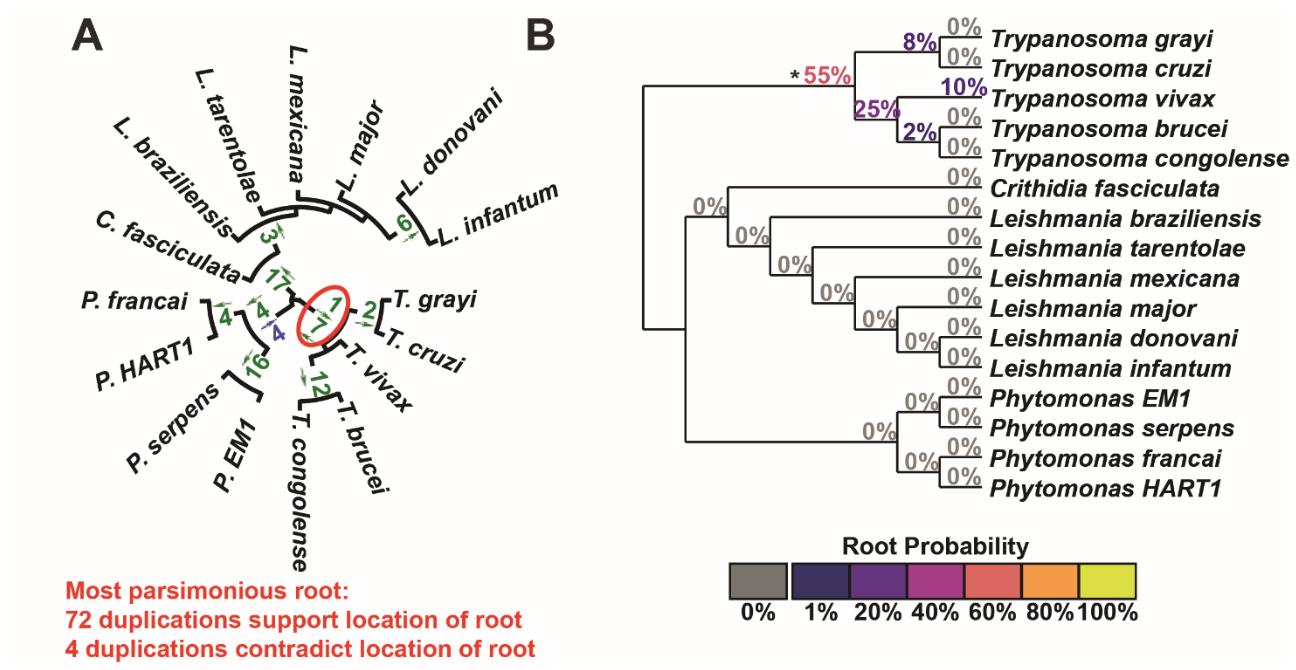
A
B
L. braziliensis
L. tarentolae
L. mexicana
L. major
L. donovani
L. infantum
C. fasciculata
P. francai
P. HART1
P. serpens
P. EM1
T. congolense
T. brucei
T. vivax
T. cruzi
T. grayi
Most parsimonious root:
72 duplications support location of root
4 duplications contradict location of root
Trypanosoma grayi
Trypanosoma cruzi
Trypanosoma vivax
Trypanosoma brucei
Trypanosoma congolense
Crithidia fasciculata
Leishmania braziliensis
Leishmania tarentolae
Leishmania mexicana
Leishmania major
Leishmania donovani
Leishmania infantum
Phytomonas EM1
Phytomonas serpens
Phytomonas francai
Phytomonas HART1
*55%
8%
25%
10%
2%
Root Probability
0%
1%
20%
40%
60%
80%
100%

Fig. S8

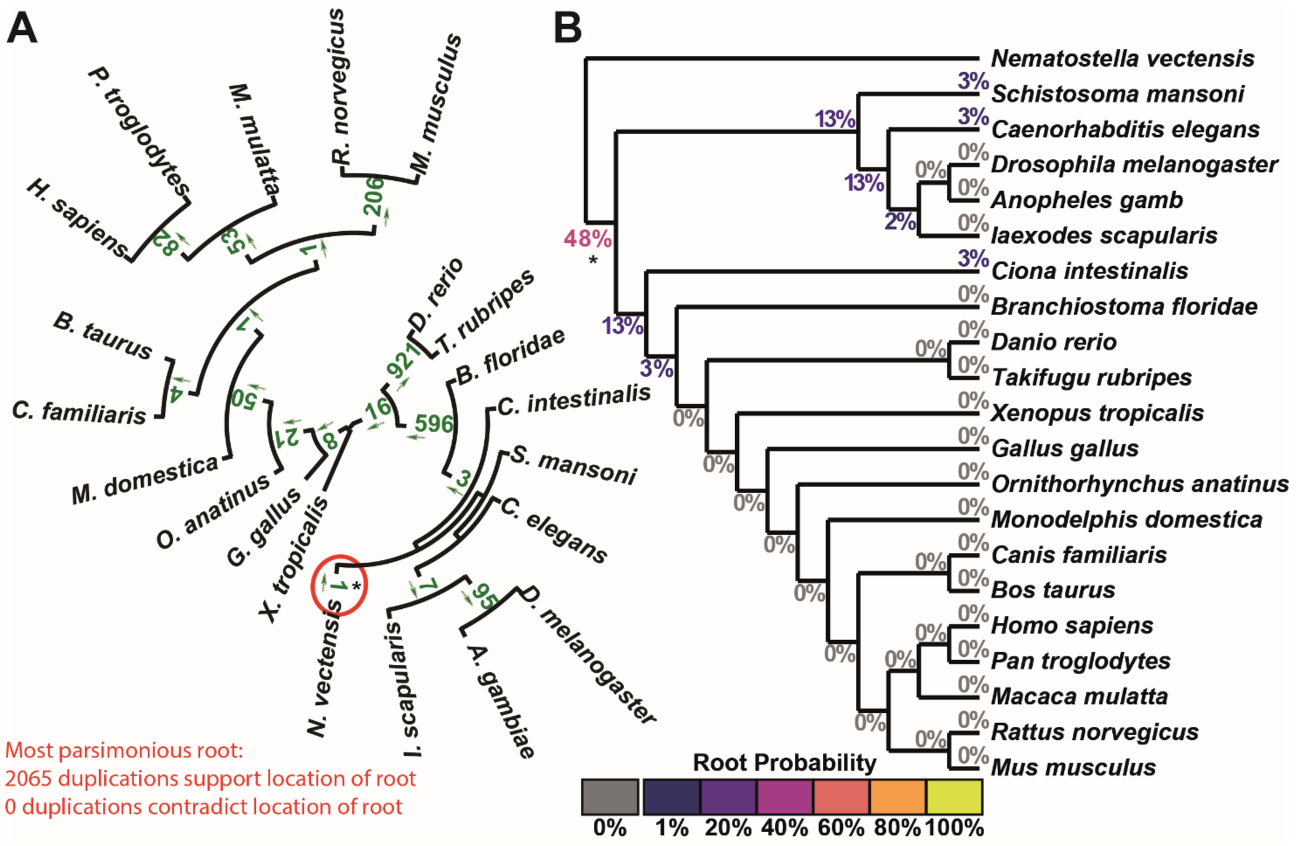
A
H. sapiens
P. troglodytes
M. mulatta
R. norvegicus
M. musculus
B. taurus
C. familiaris
M. domestica
O. anatinus
G. gallus
X. tropicalis
N. vectensis
I. scapularis
A. gambiae
D. melanogaster
C. elegans
S. mansoni
C. intestinalis
B. floridae
T. rubripes
D. rerio
28
53
206
1
1
4
50
21
8
16
921
596
3
1
7
95
Most parsimonious root:
2065 duplications support location of root
0 duplications contradict location of root
B
Nematostella vectensis
Schistosoma mansoni
Caenorhabditis elegans
Drosophila melanogaster
Anopheles gamb
Iaexodes scapularis
Ciona intestinalis
Branchiostoma floridae
Danio rerio
Takifugu rubripes
Xenopus tropicalis
Gallus gallus
Ornithorhynchus anatinus
Monodelphis domestica
Canis familiaris
Bos taurus
Homo sapiens
Pan troglodytes
Macaca mulatta
Rattus norvegicus
Mus musculus
48%
13%
13%
2%
3%
0%
Root Probability
0%
1%
20%
40%
60%
80%
100%

Fig. S9

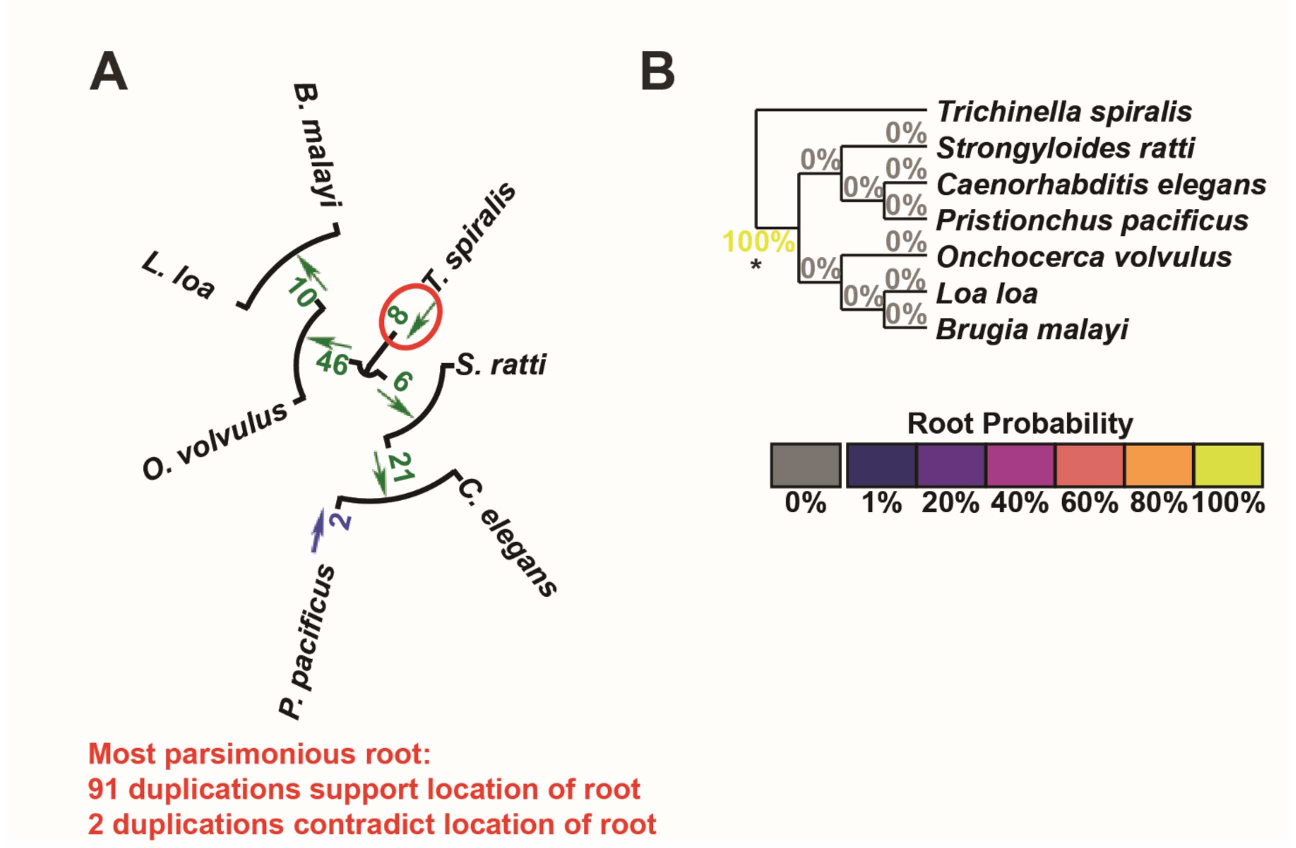
A
B. malayi
L. loa
T. spiralis
O. volvulus
S. ratti
C. elegans
P. pacificus
10
46
8
6
21
2
Most parsimonious root:
91 duplications support location of root
2 duplications contradict location of root
B
Trichinella spiralis
Strongyloides ratti
Caenorhabditis elegans
Pristionchus pacificus
Onchocerca volvulus
Loa loa
Brugia malayi
100%
*
0%
Root Probability
0%
1%
20%
40%
60%
80%
100%

Fig. S10

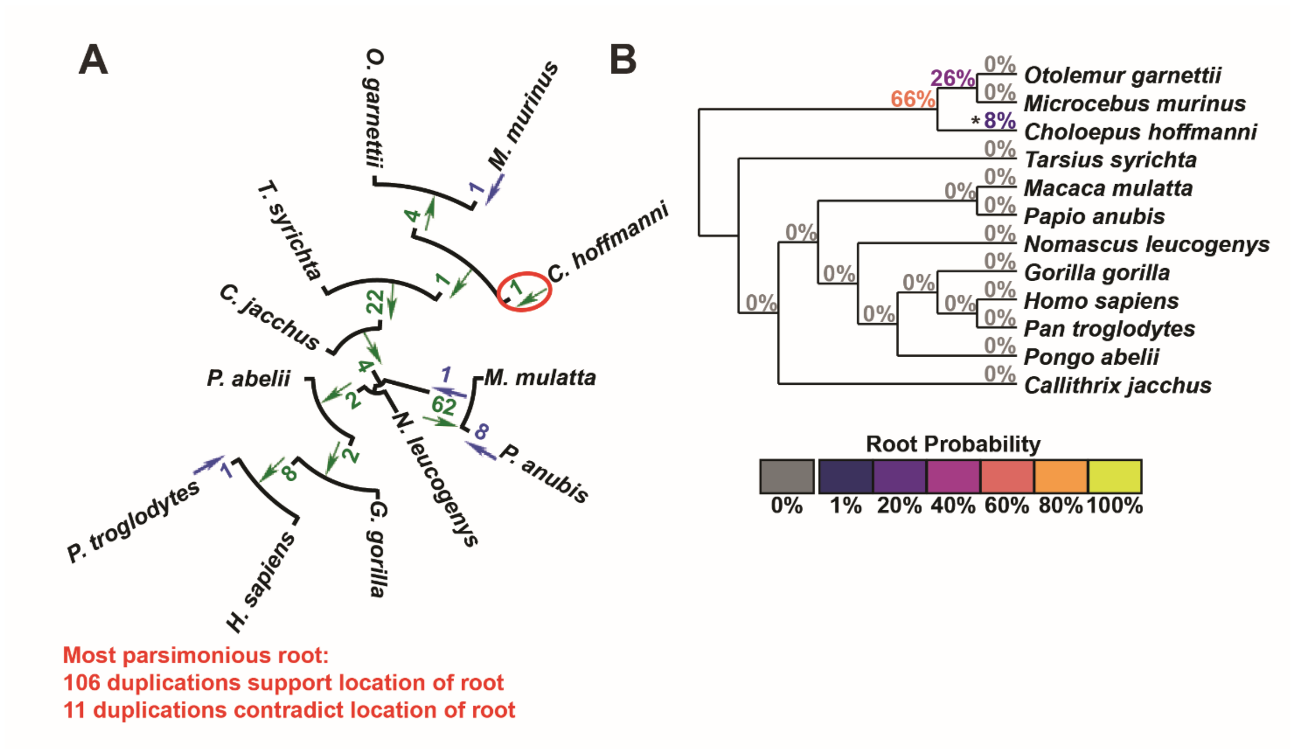
A
B
O. garnettii
M. murinus
C. hoffmanni
T. syrichta
C. jacchus
P. abelii
M. mulatta
P. anubis
N. leucogenys
G. gorilla
H. sapiens
P. troglodytes
Most parsimonious root:
106 duplications support location of root
11 duplications contradict location of root
Otolemur garnettii
Microcebus murinus
Choloepus hoffmanni
Tarsius syrichta
Macaca mulatta
Papio anubis
Nomascus leucogenys
Gorilla gorilla
Homo sapiens
Pan troglodytes
Pongo abelii
Callithrix jacchus
66%
26%
*8%
Root Probability
0% 1% 20% 40% 60% 80% 100%

Fig. S11

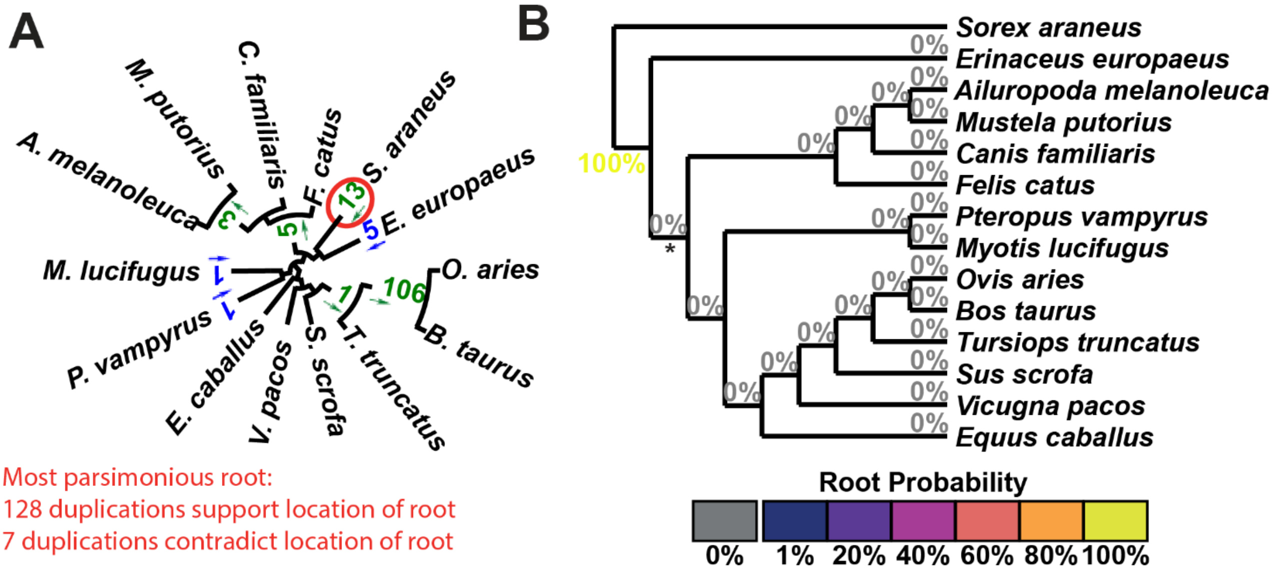
A
M. putorius
C. familiaris
F. catus
S. araneus
A. melanoleuca
E. europaeus
M. lucifugus
O. aries
P. vampyrus
B. taurus
E. caballus
V. pacos
S. scrofa
T. truncatus
3
5
13
5
1
1
1
106
Most parsimonious root:
128 duplications support location of root
7 duplications contradict location of root
B
Sorex araneus
Erinaceus europaeus
Ailuropoda melanoleuca
Mustela putorius
Canis familiaris
Felis catus
Pteropus vampyrus
Myotis lucifugus
Ovis aries
Bos taurus
Tursiops truncatus
Sus scrofa
Vicugna pacos
Equus caballus
100%
0%
*
Root Probability
0%
1%
20%
40%
60%
80%
100%

Fig. S12

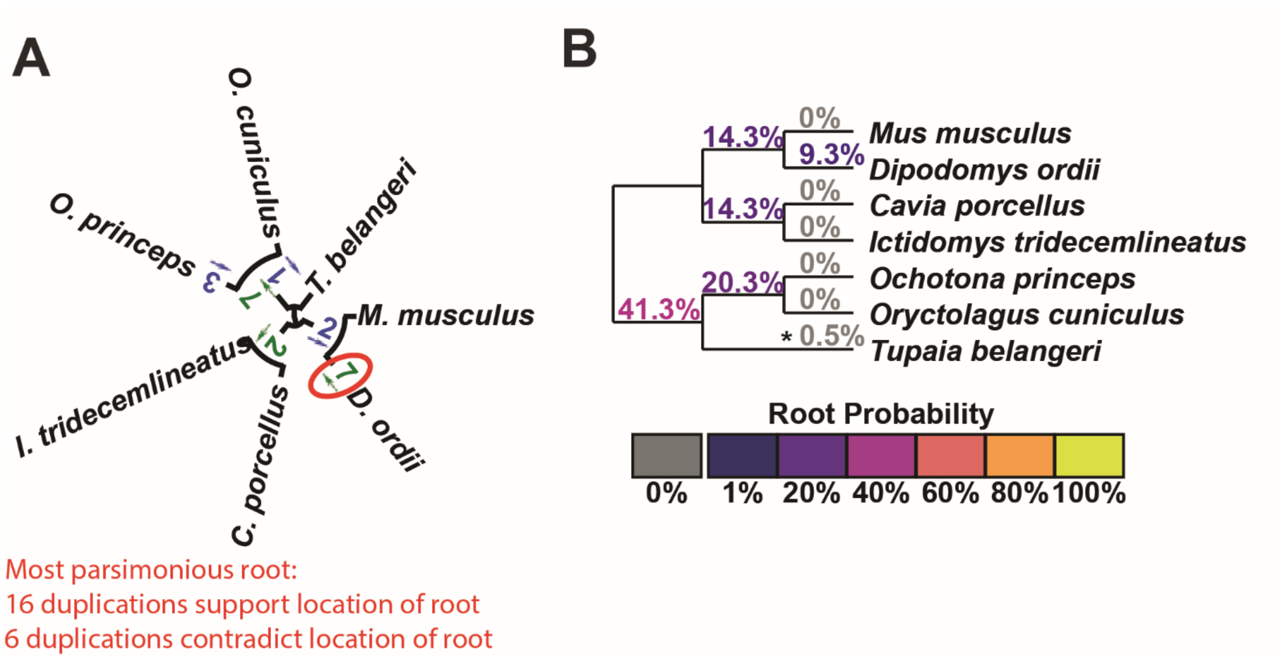
A
O. cuniculus
T. belangeri
O. princeps
M. musculus
I. tridecemlineatus
C. porcellus
D. ordii
3
1
7
2
2
7
Most parsimonious root:
16 duplications support location of root
6 duplications contradict location of root
B
0%
14.3%
Mus musculus
9.3%
Dipodomys ordii
0%
14.3%
Cavia porcellus
0%
Ictidomys tridecemlineatus
0%
20.3%
Ochotona princeps
0%
41.3%
Oryctolagus cuniculus
* 0.5%
Tupaia belangeri
Root Probability
0%
1%
20%
40%
60%
80%
100%

Fig. S13

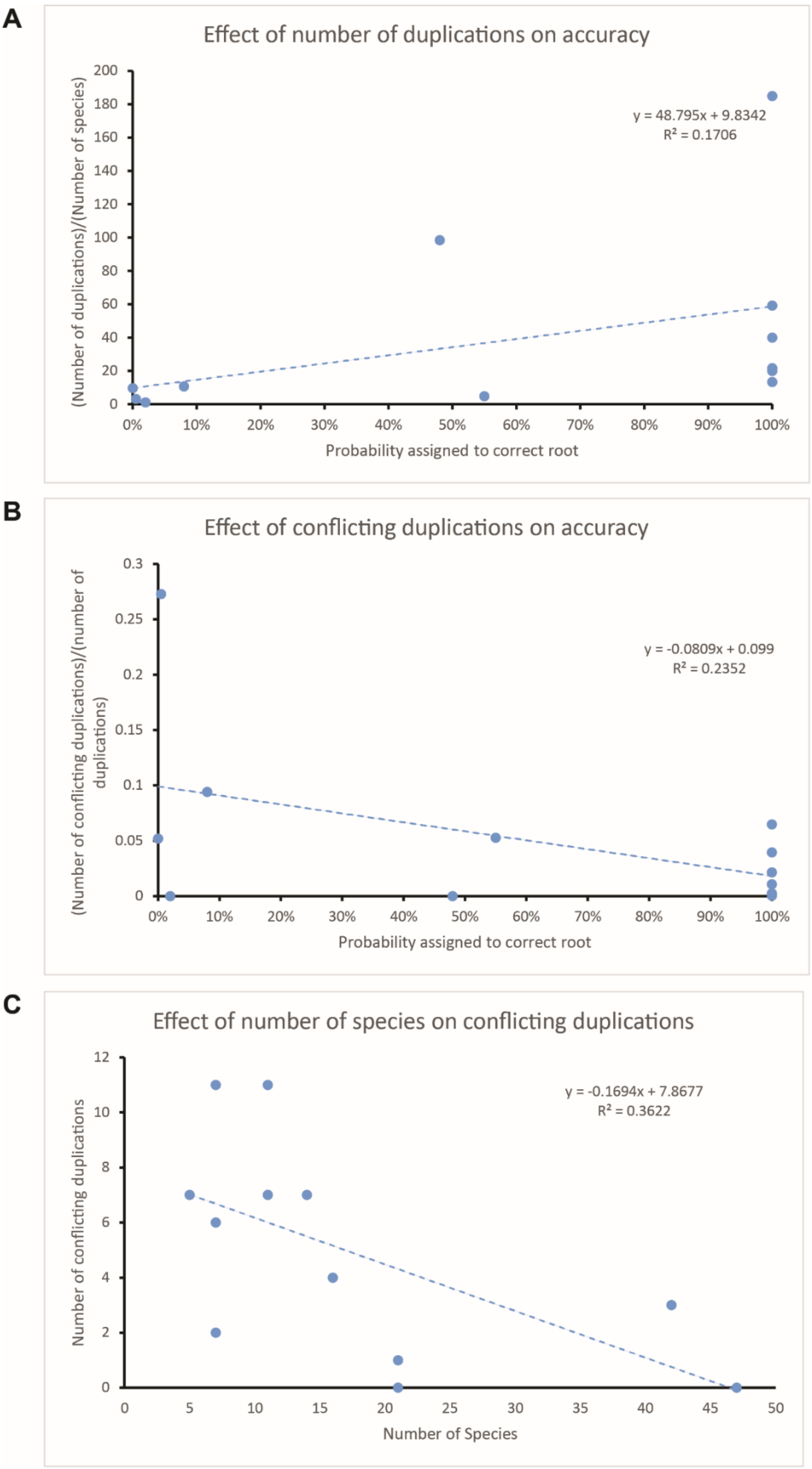
A
Effect of number of duplications on accuracy
(Number of duplications)/(Number of species)
Probability assigned to correct root
y = 48.795x + 9.8342
R² = 0.1706
B
Effect of conflicting duplications on accuracy
(Number of conflicting duplications)/(number of duplications)
Probability assigned to correct root
y = -0.0809x + 0.099
R² = 0.2352
C
Effect of number of species on conflicting duplications
Number of conflicting duplications
Number of Species
y = -0.1694x + 7.8677
R² = 0.3622

Fig. S14

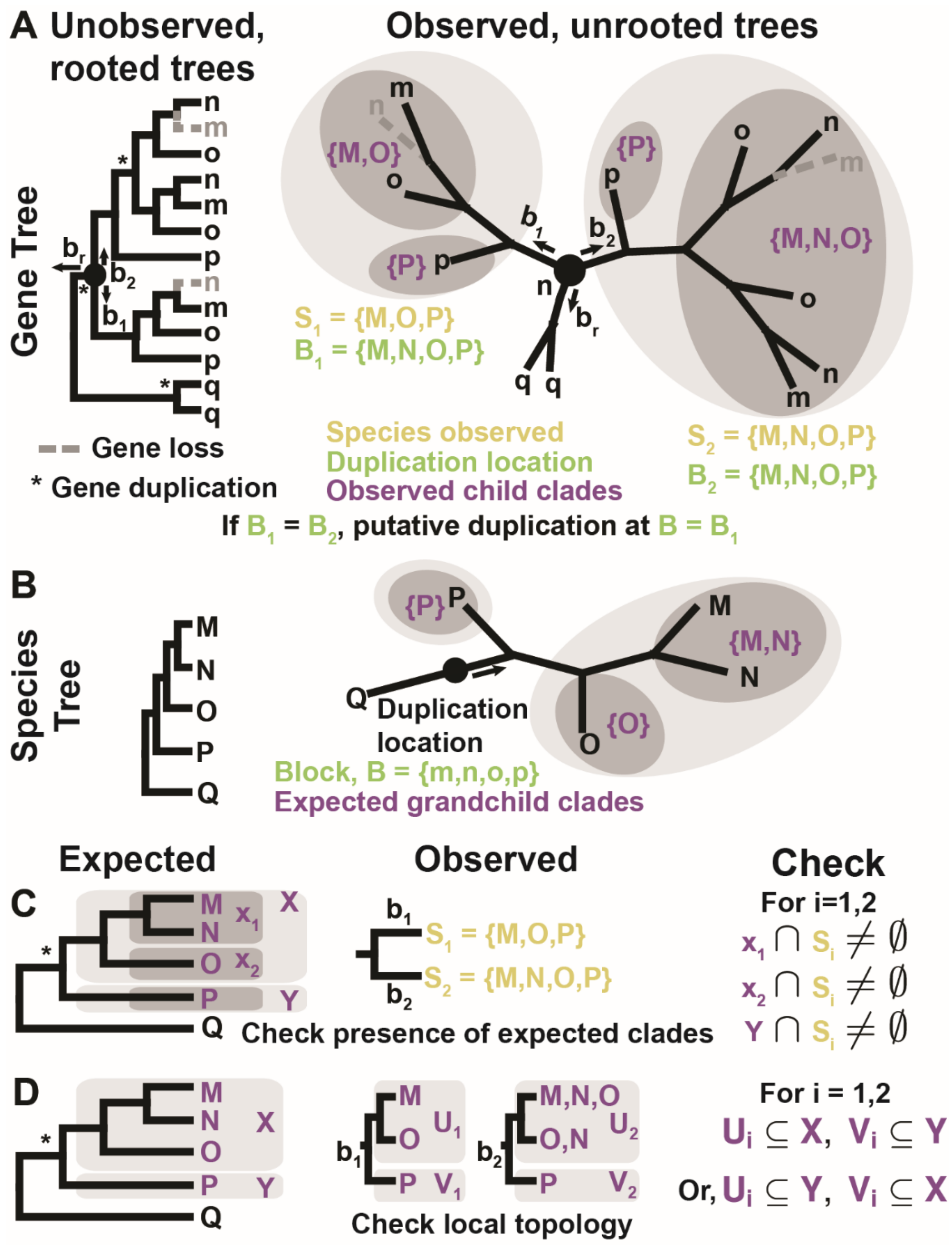
A Unobserved, rooted trees
Observed, unrooted trees
Gene Tree
Gene loss
* Gene duplication
Species observed
Duplication location
Observed child clades
If B1 = B2, putative duplication at B = B1
B
Species Tree
Duplication location
Block, B = {m,n,o,p}
Expected grandchild clades
Expected
Observed
Check
C
For i=1,2
Check presence of expected clades
D
For i = 1,2
Check local topology

Fig. S15

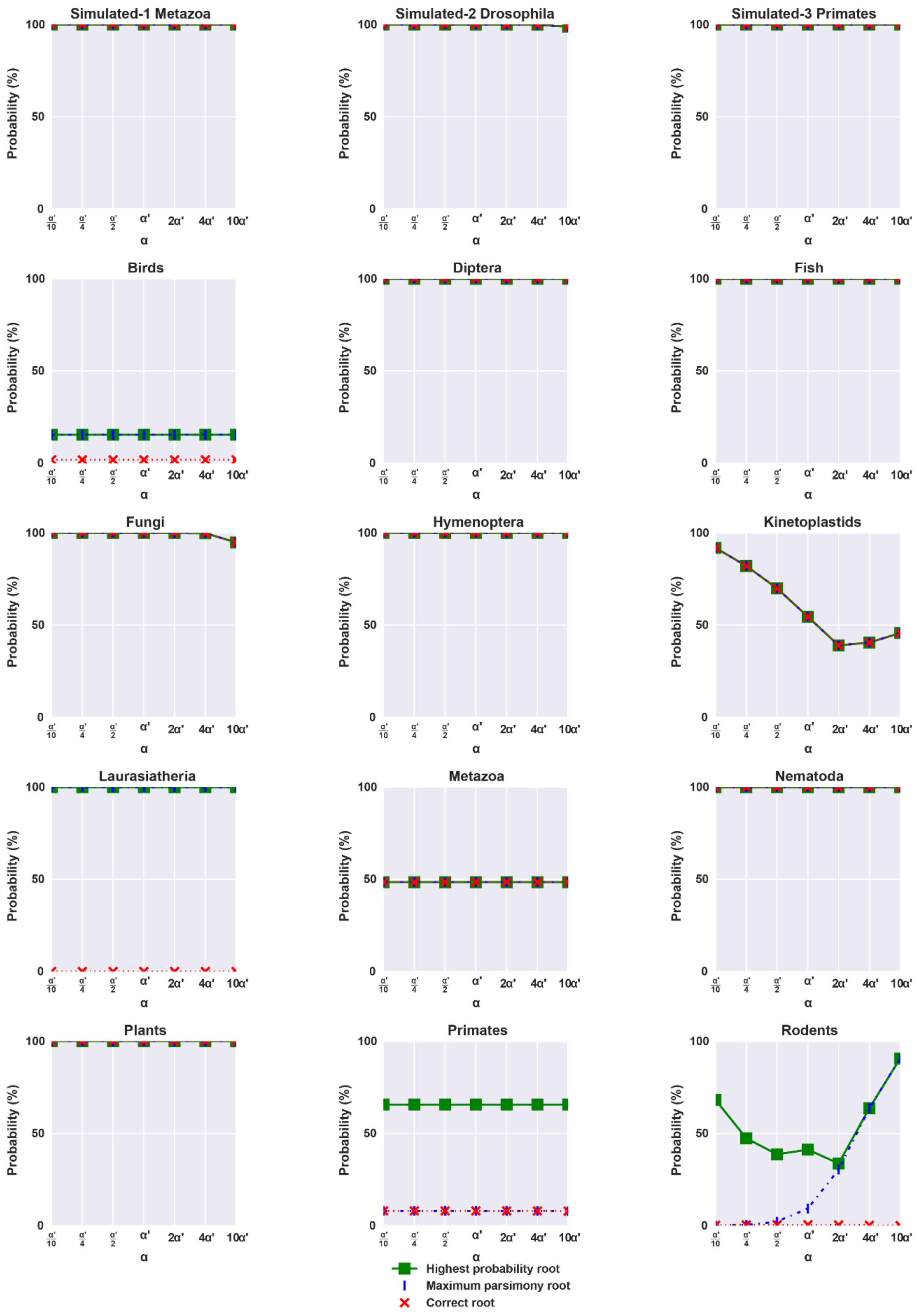
Simulated-1 Metazoa
Simulated-2 Drosophila
Simulated-3 Primates
Birds
Diptera
Fish
Fungi
Hymenoptera
Kinetoplastids
Laurasiatheria
Metazoa
Nematoda
Plants
Primates
Rodents
Probability (%)
α
Highest probability root
Maximum parsimony root
Correct root

Table S1: Summary of gene duplication prediction accuracy on simulated datasets

		STRIDE				Notung				DLCpar_search			
	**nDups**	**TP**	**FP**	**P (%)**	**R (%)**	**TP**	**FP**	**P (%)**	**R (%)**	**TP**	**FP**	**P (%)**	**R (%)**
Metazoa	1440	664	0	100.0	46.1	1347	4216	24.2	93.5	1207	85	93.4	83.8
Flies	4628	1359	1	99.9	29.3	3613	20531	15.0	78.1	3479	8624	28.7	75.2
Primates	4317	1592	1	99.9	36.8	3763	7996	32.0	87.2	3133	1620	65.9	72.6
**Total**	**10385**	**3615**	**2**	**99.9**	**34.8**	**8723**	**32743**	**21.0**	**84.0**	**7819**	**10329**	**43.0**	**75.3**

nDups = number of gene duplication events in the simulated dataset. TP = true positive. FP = false positive. P = precision. R = recall.
